# Supplementary material for: CFM: confinement force microscopy—a dynamic, precise and stable microconfiner for traction force microscopy in spatial confinement
Source: Nat Methods. 2026 Sep 14;23(10):2066–76. doi: 10.1038/s41592-026-03216-5 (PMC13645677; doi:10.1038/s41592-026-03216-5)
Supplement: Supplementary file 2 [file 41592_2026_3216_MOESM2_ESM.pdf]

nature portfolio

Corresponding author(s): Timo Betz, Fatemeh Abbasi

Last updated by author(s): Apr 20, 2026

Reporting Summary

Nature Portfolio wishes to improve the reproducibility of the work that we publish. This form provides structure for consistency and transparency in reporting. For further information on Nature Portfolio policies, see our Editorial Policies and the Editorial Policy Checklist.

Statistics

For all statistical analyses, confirm that the following items are present in the figure legend, table legend, main text, or Methods section.

n/a	Confirmed	
☐	☒	The exact sample size (*n*) for each experimental group/condition, given as a discrete number and unit of measurement
☐	☒	A statement on whether measurements were taken from distinct samples or whether the same sample was measured repeatedly
☐	☒	The statistical test(s) used AND whether they are one- or two-sided<br>*Only common tests should be described solely by name; describe more complex techniques in the Methods section.*
☒	☐	A description of all covariates tested
☐	☒	A description of any assumptions or corrections, such as tests of normality and adjustment for multiple comparisons
☐	☒	A full description of the statistical parameters including central tendency (e.g. means) or other basic estimates (e.g. regression coefficient) AND variation (e.g. standard deviation) or associated estimates of uncertainty (e.g. confidence intervals)
☐	☒	For null hypothesis testing, the test statistic (e.g. *F*, *t*, *r*) with confidence intervals, effect sizes, degrees of freedom and *P* value noted<br>*Give P values as exact values whenever suitable.*
☒	☐	For Bayesian analysis, information on the choice of priors and Markov chain Monte Carlo settings
☒	☐	For hierarchical and complex designs, identification of the appropriate level for tests and full reporting of outcomes
☐	☒	Estimates of effect sizes (e.g. Cohen's *d*, Pearson's *r*), indicating how they were calculated

Our web collection on statistics for biologists contains articles on many of the points above.

Software and code

Policy information about availability of computer code

Data collection	SlideBook was used for imaging, and a custom LabVIEW program was used to control sensor positioning and piezo-stage actuation.
Data analysis	Data analysis was performed using custom MATLAB scripts for traction-force analysis and Elastix for image registration where applicable. The custom MATLAB traction-force analysis code and the LabVIEW CFM controller program are publicly available on Zenodo (DOIs provided in the Code Availability statement and the Results.

For manuscripts utilizing custom algorithms or software that are central to the research but not yet described in published literature, software must be made available to editors and reviewers. We strongly encourage code deposition in a community repository (e.g. GitHub). See the Nature Portfolio guidelines for submitting code & software for further information.

Data

Policy information about availability of data

All manuscripts must include a data availability statement. This statement should provide the following information, where applicable:

- Accession codes, unique identifiers, or web links for publicly available datasets
- A description of any restrictions on data availability
- For clinical datasets or third party data, please ensure that the statement adheres to our policy

The authors have already provided the numerical source data underlying the figures and can provide any additional data upon reasonable request.

Research involving human participants, their data, or biological material

Policy information about studies with human participants or human data. See also policy information about sex, gender (identity/presentation), and sexual orientation and race, ethnicity and racism.

Reporting on sex and gender	Not applicable; this study did not involve human participants, human participant data, or prospectively collected human biological material. Established cell lines are reported separately in the “Eukaryotic cell lines” section.
Reporting on race, ethnicity, or other socially relevant groupings	Not applicable; this study did not involve human participants or human participant data.
Population characteristics	Not applicable; this study did not involve human participants
Recruitment	Not applicable; this study did not involve human participants.
Ethics oversight	Not applicable; this study did not involve human participants, human participant data, or prospectively collected human biological material.

Note that full information on the approval of the study protocol must also be provided in the manuscript.

Field-specific reporting

Please select the one below that is the best fit for your research. If you are not sure, read the appropriate sections before making your selection.

☒ Life sciences ☐ Behavioural & social sciences ☐ Ecological, evolutionary & environmental sciences

For a reference copy of the document with all sections, see nature.com/documents/nr-reporting-summary-flat.pdf

Life sciences study design

All studies must disclose on these points even when the disclosure is negative.

Sample size	To demonstrate the method, we used sample sizes sufficient to assess reproducibility, with cells typically measured from at least three independent samples collected on three different days. Sample sizes were guided by the initial variance and the observed data distribution.
Data exclusions	Data were excluded if the cellular phenotype raised concerns about viability. Data were also excluded if the analysis software failed to load or process the files, if imaging was interrupted (resulting in discontinuities), or if the analysis pipeline did not return a valid output.
Replication	All data used for statistical test had at least 3 replicates
Randomization	Sample were chosen randomly.
Blinding	Blinding was not necessary, as all analyses were performed using automated pipelines.

Reporting for specific materials, systems and methods

We require information from authors about some types of materials, experimental systems and methods used in many studies. Here, indicate whether each material, system or method listed is relevant to your study. If you are not sure if a list item applies to your research, read the appropriate section before selecting a response.

Materials & experimental systems

n/a	Involved in the study	
☐	☐	Antibodies
☐	☒	Eukaryotic cell lines
☐	☐	Palaeontology and archaeology
☐	☒	Animals and other organisms
☐	☐	Clinical data
☐	☐	Dual use research of concern
☐	☐	Plants

Methods

n/a	Involved in the study	
☐	☐	ChIP-seq
☐	☐	Flow cytometry
☐	☐	MRI-based neuroimaging

Antibodies

Antibodies used	Not applicable.
Validation	*Describe the validation of each primary antibody for the species and application, noting any validation statements on the manufacturer's website, relevant citations, antibody profiles in online databases, or data provided in the manuscript.*

Eukaryotic cell lines

Policy information about cell lines and Sex and Gender in Research

Cell line source(s)	HeLa and CT26 cell lines were kind gifts from Danijela Vignjević (Institut Curie, Paris). C2C12 cells were obtained from an established laboratory stock. HoxB8 cells were a gift from Johannes Roth (Institute of Immunology, Münster).
Authentication	C2C12, HeLa and CT26 cells were authenticated by phenotype, based on their characteristic morphology and growth behavior. HoxB8 cells were authenticated by phenotype through their differentiation into neutrophils.
Mycoplasma contamination	All cell lines used in this study were tested frequently for mycoplasma contamination and were negative.
Commonly misidentified lines (See ICLAC register)	HeLa is listed in the ICLAC Register as a commonly misidentified/cross-contaminating cell line; in this study, HeLa cells were used intentionally as an established human cervical cancer model, and their identity as HeLa was not in question. The other cell lines used in this study were not listed as models of disputed identity on the basis of the information available in the current ICLAC Register.

Palaeontology and Archaeology

Specimen provenance	Not applicable.
Specimen deposition	*Indicate where the specimens have been deposited to permit free access by other researchers.*
Dating methods	*If new dates are provided, describe how they were obtained (e.g. collection, storage, sample pretreatment and measurement), where they were obtained (i.e. lab name), the calibration program and the protocol for quality assurance OR state that no new dates are provided.*

☐ Tick this box to confirm that the raw and calibrated dates are available in the paper or in Supplementary Information.

Ethics oversight	*Identify the organization(s) that approved or provided guidance on the study protocol, OR state that no ethical approval or guidance was required and explain why not.*

Note that full information on the approval of the study protocol must also be provided in the manuscript.

Animals and other research organisms

Policy information about studies involving animals; ARRIVE guidelines recommended for reporting animal research, and Sex and Gender in Research

Laboratory animals	Drosophila melanogaster embryos from the sqhAX3; sqh-Sqh-GFP fly line (BDSC 57144) were used in this study.
Wild animals	No wild animals were used in this study. All Drosophila melanogaster embryos were obtained from a laboratory fly stock (sqhAX3; sqh-Sqh-GFP, BDSC 57144).
Reporting on sex	Sex was not determined at the embryonic stage.
Field-collected samples	No field-collected samples were used in this study. All biological material was obtained from established laboratory stocks or cell lines.
Ethics oversight	Not applicable; this study involved established cell lines and Drosophila melanogaster embryos from laboratory stocks and did not involve human participants or regulated vertebrate animal experiments.

Note that full information on the approval of the study protocol must also be provided in the manuscript.

Clinical data

Policy information about clinical studies

All manuscripts should comply with the ICMJE guidelines for publication of clinical research and a completed CONSORT checklist must be included with all submissions.

Clinical trial registration	Not applicable.
Study protocol	Not applicable
Data collection	Not applicable
Outcomes	Not applicable

Dual use research of concern

Policy information about dual use research of concern

Hazards

Could the accidental, deliberate or reckless misuse of agents or technologies generated in the work, or the application of information presented in the manuscript, pose a threat to:

No	Yes	
☒	☐	Public health
☒	☐	National security
☒	☐	Crops and/or livestock
☒	☐	Ecosystems
☒	☐	Any other significant area

Experiments of concern

Does the work involve any of these experiments of concern:

No	Yes	
☒	☐	Demonstrate how to render a vaccine ineffective
☒	☐	Confer resistance to therapeutically useful antibiotics or antiviral agents
☒	☐	Enhance the virulence of a pathogen or render a nonpathogen virulent
☒	☐	Increase transmissibility of a pathogen
☒	☐	Alter the host range of a pathogen
☒	☐	Enable evasion of diagnostic/detection modalities
☒	☐	Enable the weaponization of a biological agent or toxin
☒	☐	Any other potentially harmful combination of experiments and agents

Plants

Seed stocks	Not applicable.
Novel plant genotypes	Not applicable.
Authentication	Not applicable.

ChIP-seq

Data deposition

☐ Confirm that both raw and final processed data have been deposited in a public database such as GEO.

☐ Confirm that you have deposited or provided access to graph files (e.g. BED files) for the called peaks.

Data access links<br>*May remain private before publication.*	Not applicable.
Files in database submission	Not applicable.
Genome browser session<br>(e.g. UCSC)	Not applicable.

Methodology

Replicates	Not applicable.
Sequencing depth	Not applicable.
Antibodies	Not applicable.
Peak calling parameters	Not applicable.
Data quality	Not applicable.
Software	Not applicable.

Flow Cytometry

Plots

Confirm that:

☐ The axis labels state the marker and fluorochrome used (e.g. CD4-FITC).

☐ The axis scales are clearly visible. Include numbers along axes only for bottom left plot of group (a 'group' is an analysis of identical markers).

☐ All plots are contour plots with outliers or pseudocolor plots.

☐ A numerical value for number of cells or percentage (with statistics) is provided.

Methodology

Sample preparation	Not applicable.
Instrument	Not applicable.
Software	Not applicable.
Cell population abundance	Not applicable.
Gating strategy	Not applicable.

☐ Tick this box to confirm that a figure exemplifying the gating strategy is provided in the Supplementary Information.

Magnetic resonance imaging

Experimental design

Design type	Not applicable.
Design specifications	Not applicable.
Behavioral performance measures	Not applicable.

Acquisition

Imaging type(s)	Not applicable.
Field strength	Not applicable.
Sequence & imaging parameters	Not applicable.
Area of acquisition	Not applicable.

Diffusion MRI ☐ Used ☐ Not used

Preprocessing

Preprocessing software	Not applicable.
Normalization	Not applicable.
Normalization template	Not applicable.
Noise and artifact removal	Not applicable.
Volume censoring	v

Statistical modeling & inference

Model type and settings	Not applicable.
Effect(s) tested	Not applicable.

Specify type of analysis: ☐ Whole brain ☐ ROI-based ☐ Both

Statistic type for inference (See Eklund et al. 2016)	Not applicable.
Correction	Not applicable.

Models & analysis

n/a	Involved in the study
☐	☐ Functional and/or effective connectivity
☐	☐ Graph analysis
☐	☐ Multivariate modeling or predictive analysis

Functional and/or effective connectivity	Not applicable.
Graph analysis	Not applicable.
Multivariate modeling and predictive analysis	Not applicable.
