# Supplementary material for: CFM: confinement force microscopy—a dynamic, precise and stable microconfiner for traction force microscopy in spatial confinement
Source: Nat Methods. 2026 Sep 14;23(10):2066–76. doi: 10.1038/s41592-026-03216-5 (PMC13645677; doi:10.1038/s41592-026-03216-5)
Supplement: Supplementary file 3 [file 41592_2026_3216_MOESM3_ESM.pdf]

nature portfolio

Peer Review File

CFM: Confinement Force Microscopy-a dynamic, precise and stable microconfiner for traction force microscopy in spatial confinement

Corresponding Author: Professor Timo Betz

This file contains all editorial decision letters in order by version, followed by all author rebuttals in order by version.

A version of this paper was originally rejected for publication by Nature Methods, however that decision was reconsidered after appeal by the authors.

Version 0:

Decision Letter:

16th Nov 2023

Dear Timo,

Your Article entitled "CFM: Confinement Force Microscopy-a dynamic, precise and stable microconfiner for traction force microscopy in spatial confinement" has now been seen by 2 reviewers, whose comments are attached. While they find your work of some potential interest, they have raised concerns which in our view are sufficiently important that they preclude publication of the work in Nature Methods.

We will consider looking at a revised manuscript only if further experimental data allow you to address all the major criticisms of the reviewers (unless, of course, something similar has by then been accepted at Nature Methods or appeared elsewhere). This includes submission or publication of a portion of this work somewhere else.

The required new experiments and data include, but are not limited to, evidence highlighting the technical advance represented by CFM as well as strong demonstrations on how this method might enable novel biological insights. We hope you understand that until we have read the revised paper in its entirety we cannot promise that it will be sent back for peer-review.

If you are interested in revising this manuscript for submission to Nature Methods in the future, please contact me to discuss your appeal before making any revisions. Otherwise, we hope that you find the reviewers' comments helpful when preparing your paper for submission elsewhere.

Sincerely,
Madhura

Madhura Mukhopadhyay, PhD
Senior Editor
Nature Methods

Reviewers' Comments:

Reviewer #1:
Remarks to the Author:
In this article, Abbasi and colleagues propose a novel confiner device that allows a precise control of the confinement height combined with simultaneous measurement of lateral and normal forces (3D traction force). The strength of the device is the combination of these three features, as they were all achieved previously independantly of each other, or in combination of two

of them, but not the three. The device is well caracterised and rather simple. Most of the article is dedicated to short proofs of principle for applications of the method to a variety of contexts, from single cells to developping embryos or cell aggregates. This makes it clear that the device will be usefull for a large set of researchers. The drawback is that none of the application is really pushed far enough to convincingly make the point of the unique capacity of this method to bring novel insights. While the method is a clear technical breakthrough, the article remains, in its present form, too superficial in the applications it proposes for the method.

Main concerns:
1) Writing: the current style of the article is, being a bit caricatural, a little like an advertisement for a new product, combined with some application notes. I think this should be changed. In the introduction, listing the previous confinement devices is useful, but the way their drawbacks is presented against all the advantages of the new method is not so interesting and often a bit biased if not almost incorrect (like when the authors say that the new method 'simulate the in vivo mechanical and chemical environments experienced by cells and tissue' - this is a clear overstatement!). One can propose a table for such comparisons, but for the intro, it is more interesting to present what has been achieved so far and the important discoveries made with confinement devices, to show that this is an interesting topic for cell biology, and then simply present the novel possibilities brought by the present device. ANd it is self-evident that these new implementations were not possible with previous devices. The focus being obviously the combination of confinement with a 3D force measure and what can be done with this combination.

2) The choice of the authors to show many possible applications is at the expense of going far enough in any of them. I think it is not a very good choice. Maybe the authors could remove some (or just mention them in a sup figure) and spend more time on showing specifically what new can be learned from the combination of confinement with 3D force measures. In particular, I think that what needs to be shown is that knowing both the normal and lateral stress makes a difference in the understanding of the cell response. For example, relaxation can be well studied with just the normal or just the lateral forces, especially if they are similar in both axis (is it the case?). Similarly, the change in the force exerted by the cells as the confinement increases, and for various confinement levels, can be studied independantly for the normal force (with an AFM, as previously published by several teams), or only lateraly with regular 2D traction force. So the authors should show an application when measuring forces on both axis on the same cell makes a difference.

2) Taking specifically each proposed application, there are a few obvious points that the authors should adress to substantiate the results that they report:

a) on the confinement of single HeLA cells and neutrophiles (figures 2 and 3): several reports have shown the induction of persistent blebbing upon confinement, or at least a higher contracility (in immune cells). Did the authors also observe that? For example, is the plateau in the stress increase for the very low confinement, on neutrophiles, due to blebbing, which could reduce the stress? And how much of the stress observed is due to the cell cortex contractility (comparing with cells treated with Y27632 for example)? It is also not clear whether the stress increase is an active response (in neutrophiles), or rather the absense of stress increase (in HeLa). As the authors show, a passive object shows an increase in stress as it gets deformed. Is it what is observed on neutrophiles before the plateau for the very low confinement? Or do they increase the stress more, suggesting an increase in contractility, as reported for other cells (including immune cells) before? And for Hela cells, is the absence of increase due to blebbing? Others, using an AFM device, have reported an increase in stress, more similar to the 'passive' response, until the cell eventually increases contractility, leading to blebbing. Could the difference also be due to the adhesive versus non adhesive state of cells (neutrophiles, or the Hela studied in previous studies, are not adhesive or on non adhesive substrates, while here cells are adhesive, especially the HeLa cells). Can the authors modulate the adhesiveness of the substrate?
This is a lot of points to adress, but a complete study on one aspect is more interesting, even for a methods paper, than a lot of rather superficially studied examples.

b)The durotaxis example is really great and appears also more novel and more unique to the possibilities offered by the presented method, because of the two gels. One aspect in which it could be completed is to also report the inverse situation, in which cells are attached initially to a soft roof and then transit to a stiffer bottom gel, which would allow to report the forces on the stiff substrate towardds which cells migrate, and complement what the authors report on the soft gel side, that the cells leave. In addition, it would be usefull to document the state of focal adhesions during the process, to correlate with the forces. FInally, a discussion of how these results compare to what was reported on forces that cells exert when spreading between two plates (for example from the Asnacios lab) would be interesting.

c) What is reported for the oscillating constraint appears very preliminary. It is just the feasability, and even that is not really investigated (how fast can it be done while measuring forces for example?). The interpretation of the result is very qualitative for such a quantitative output. Maybe, for this aspect, just studying in more details one dynamic confinement step would be more interesting (relaxation, etc...) and give the opportunity to focus on what the XYZ force measure brings: for example is relaxation the same in both axis?

d) the application on the drosophila embryo is even less detailed and is just showing that the embryo survives the growth in the device. This is a bt anecdotical.

e) This part is interesting but it is not clear at all in which sense it is specific to this device. It seems that here only the normal stress is exploited, which means that this could be done with any classical method (and it has been done indeed).

Reviewer #2:
Remarks to the Author:
A. Key results: The authors report confinement force microscopy. This method allows to confine cells, spheroids, organoids, embryos into micrometer spacings while at the same time measuring traction forces. It can be combined with fluorescence microscopy, so that also biological changes can be recorded.

B. Originality: The method is not intrinsically novel. It is certainly a more precise version compared to previous approaches. However, having in mind the following references, the general strategy of the approach does not appear novel enough for me to justify publication in Nature methods. For example, the following references, which also combined confinement with (traction) force measurements, have not been mentioned in the manuscript:
• Raman et al., Probing cell traction forces in confined microenvironments (2013): https://pubs.rsc.org/en/content/articlelanding/2013/LC/c3lc50802a
• Hui et al., Cell traction force in a confined microenvironment with double-sided micropost arrays (2019): https://pubs.rsc.org/en/content/articlelanding/2019/RA/C8RA10170A
• Brunner et al., Cell migration through small gaps (2006): https://link.springer.com/article/10.1007/s00249-006-0079-1
In addition, also cytocompression is not new and the simple combination with traction force microscopy is a nice and biophysically interesting aspect to study, but again in my opinion this does not justify publication in Nature Methods:
• There are several reports on cytocompression assays, including Ofek et al., 2009: https://www.cell.com/fulltext/S0006-3495(09)01301-0

C. Data and methodology: The data are very well prepared, the methodology is an improved version of previously published similar approaches. The quality of the data and of the presentation is excellent and the device is more precise than others before, but the innovation is limited compared the previous ones.

D. Statistics: Appropriate.

E. Conclusions: They are robust, but I am missing a discussion in the context of the abovementioned literature.

F. Suggestions: I do not see that the approach is novel enough for publication in Nature Methods. It is a great paper, and an improvement of previous approaches, but not original enough.

G. References: See missing references above.

H. Clarity: Appropriate.

** For Nature Portfolio general information and news for authors, see http://npg.nature.com/authors.

Version 1:

Decision Letter:

7th Dec 2023

Dear Timo,

Thank you for your letter asking us to reconsider our decision on your Article, "CFM: Confinement Force Microscopy-a dynamic, precise and stable microconfiner for traction force microscopy in spatial confinement". After careful consideration we have decided that we are willing to consider a revised version of your manuscript as outlined in your plan. Please make sure the revision highlights the technical advance and novelty of your method with a strong application (happy to set up a chat about this).

I also wanted to tell you that once we have had a chance to assess the revision, we will assign a new referee since reviewers 1 and 2 had contradictory opinions on the novelty of the method.

When revising your paper:

* include a point-by-point response to our referees and to any editorial suggestions

* please underline/highlight any additions to the text or areas with other significant changes to facilitate review of the revised manuscript

* address the points listed described below to conform to our open science requirements

* ensure it complies with our general format requirements as set out in our guide to authors at www.nature.com/naturemethods

* resubmit all the necessary files electronically by using the link below to access your home page

Link Redacted

Note: This URL links to your confidential home page and associated information about manuscripts you may have submitted, or that you are reviewing for us. If you wish to forward this email to co-authors, please delete the link to your homepage.

We hope to receive your revised paper within 8 weeks weeks. If you cannot send it within this time, please let us know. In this event, we will still be happy to reconsider your paper at a later date so long as nothing similar has been accepted for publication at Nature Methods or published elsewhere.

OPEN SCIENCE REQUIREMENTS

REPORTING SUMMARY AND EDITORIAL POLICY CHECKLISTS
When revising your manuscript, please submit reporting summary and editorial policy checklists.

Reporting summary: https://www.nature.com/documents/nr-reporting-summary.zip
Editorial policy checklist: https://www.nature.com/documents/nr-editorial-policy-checklist.zip

If your paper includes custom software, we also ask you to complete a supplemental reporting summary.

Software supplement: https://www.nature.com/documents/nr-software-policy.pdf

Please submit these with your revised manuscript. They will be available to reviewers to aid in their evaluation if the paper is re-reviewed. If you have any questions about the checklist, please see http://www.nature.com/authors/policies/availability.html or contact me.

Please note that these forms are dynamic 'smart pdfs' and must therefore be downloaded and completed in Adobe Reader. We will then flatten them for ease of use by the reviewers. If you would like to reference the guidance text as you complete the template, please access these flattened versions at http://www.nature.com/authors/policies/availability.html.

DATA AVAILABILITY
Please include a "Data availability" subsection in the Online Methods. This section should inform readers about the availability of the data used to support the conclusions of your study, including accession codes to public repositories, references to source data that may be published alongside the paper, unique identifiers such as URLs to data repository entries, or data set DOIs, and any other statement about data availability. At a minimum, you should include the following statement: "The data that support the findings of this study are available from the corresponding author upon request", describing which data is available upon request and mentioning any restrictions on availability. If DOIs are provided, please include these in the Reference list (authors, title, publisher (repository name), identifier, year). For more guidance on how to write this section please see: http://www.nature.com/authors/policies/data/data-availability-statements-data-citations.pdf

CODE AVAILABILITY
Please include a "Code Availability" subsection in the Online Methods which details how your custom code is made available. Only in rare cases (where code is not central to the main conclusions of the paper) is the statement "available upon request" allowed (and reasons should be specified).

We request that you deposit code in a DOI-minting repository such as Zenodo, Gigantum or Code Ocean and cite the DOI in the Reference list. We also request that you use code versioning and provide a license.

For more information on our code sharing policy and requirements, please see: https://www.nature.com/nature-research/editorial-policies/reporting-standards#availability-of-computer-code

MATERIALS AVAILABILITY
As a condition of publication in Nature Methods, authors are required to make unique materials promptly available to others without undue qualifications.

Authors reporting new chemical compounds must provide chemical structure, synthesis and characterization details. Authors reporting mutant strains and cell lines are strongly encouraged to use established public repositories.

More details about our materials availability policy can be found at https://www.nature.com/nature-portfolio/editorial-policies/reporting-standards#availability-of-materials

SUPPLEMENTARY PROTOCOL
To help facilitate reproducibility and uptake of your method, we ask you to prepare a step-by-step Supplementary Protocol for the method described in this paper. We encourage authors to share their step-by-step experimental protocols on a protocol sharing platform of their choice and report the protocol DOI in the reference list. Nature Portfolio 's Protocol Exchange is a free-to-use and open resource for protocols; protocols deposited in Protocol Exchange are citable and can be linked from the

published article. More details can found at www.nature.com/protocolexchange/about.

ORCID
Nature Methods is committed to improving transparency in authorship. As part of our efforts in this direction, we are now requesting that all authors identified as 'corresponding author' on published papers create and link their Open Researcher and Contributor Identifier (ORCID) with their account on the Manuscript Tracking System (MTS), prior to acceptance. This applies to primary research papers only. ORCID helps the scientific community achieve unambiguous attribution of all scholarly contributions. You can create and link your ORCID from the home page of the MTS by clicking on 'Modify my Springer Nature account'. For more information please visit please visit www.springernature.com/orcid.

We look forward to hearing from you soon.

Sincerely,
Madhura

Madhura Mukhopadhyay, PhD
Senior Editor
Nature Methods

Version 2:

Decision Letter:

26th Nov 2025

Dear Timo,

Your Article, "CFM: Confinement Force Microscopy-a dynamic, precise and stable microconfiner for traction force microscopy in spatial confinement", has now been seen by 2 reviewers. As you will see from their comments below, although the reviewers find your work of considerable potential interest, they have raised a number of concerns. We continue to be interested in potentially publishing your manuscript in Nature Methods, but would like to evaluate your response to these concerns before we reach a final decision on publication.

We therefore invite you to revise your manuscript to address all these concerns especially those raised by Reviewer 2. We also think it would be very important to add a stronger application to really showcase how CFM enables analysis of mechanical pressure in cellular environments.

We are committed to providing a fair and constructive peer-review process. Do not hesitate to contact us if there are specific requests from the reviewers that you believe are technically impossible or unlikely to yield a meaningful outcome.

When revising your paper:

* include a point-by-point response to the reviewers and to any editorial suggestions

* please underline/highlight any additions to the text or areas with other significant changes to facilitate review of the revised manuscript

* address the points listed described below to conform to our open science requirements

* ensure it complies with our general format requirements as set out in our guide to authors at www.nature.com/naturemethods

* resubmit all the necessary files by using the link below to access your home page

Link Redacted

Note: This URL links to your confidential account and associated information about manuscripts you may have submitted, or that you are reviewing for us. If you wish to forward this email to co-authors, please delete this link.

We hope to receive your revised paper within three months. If you are substantially delayed, please let us know. In this event, we will still be happy to reconsider your paper at a later date so long as nothing similar has been accepted for publication at Nature Methods or published elsewhere.

OPEN SCIENCE REQUIREMENTS

REPORTING SUMMARY
When revising your manuscript, please update your reporting summary.

Reporting summary: https://www.nature.com/documents/nr-reporting-summary.zip

If your paper includes custom software, we also ask you to complete a supplemental reporting summary.

Software supplement: https://www.nature.com/documents/nr-software-policy.pdf

Please submit these with your revised manuscript. They will be available to reviewers to aid in their evaluation if the paper is re-reviewed. If you have any questions about the checklist, please see http://www.nature.com/authors/policies/availability.html or contact me.

Please note that these forms are dynamic 'smart pdfs' and must therefore be downloaded and completed in Adobe Reader. We will then flatten them for ease of use by the reviewers. If you would like to reference the guidance text as you complete the template, please access these flattened versions at http://www.nature.com/authors/policies/availability.html.

For any revision that includes light microscopy data, we ask our authors to please include a completed light microscopy reporting table [https://www.nature.com/documents/Light_microscopy_reporting_table.xlsx] to ensure the methods are described thoroughly. The table will be available to reviewers and ultimately published should the manuscript be accepted at the journal.

EXTENDED DATA FIGURES
When re-submitting your manuscript, please ensure that any supplementary figures and tables that are crucial to the manuscript's conclusions are converted into Extended Data figures and tables to increase visibility of these data. Extended Data figures and tables are online-only (present in the online PDF and full-text HTML versions of the paper), peer-reviewed display items that provide essential background to the article but are not included in the main article due to space constraints. A maximum of ten Extended Data display items (figures and tables) is permitted.

DATA AVAILABILITY
We strongly encourage you to deposit all new data associated with the paper in a persistent repository where they can be freely and enduringly accessed. We recommend submitting the data to discipline-specific and community-recognized repositories; a list of repositories is provided here: http://www.nature.com/sdata/policies/repositories

All novel DNA and RNA sequencing data, protein sequences, genetic polymorphisms, linked genotype and phenotype data, gene expression data, macromolecular structures, and proteomics data must be deposited in a publicly accessible database, and accession codes and associated hyperlinks must be provided in the "Data Availability" section.

For papers containing bioimaging data, we strongly recommend depositing the data to Bioimage Archive (https://www.ebi.ac.uk/bioimage-archive/). Associated accession codes and hyperlinks should be provided in the "Data Availability" section.

Refer to our data policies here: https://www.nature.com/nature-research/editorial-policies/reporting-standards#availability-of-data

To further increase transparency, we encourage you to provide, in tabular form, the data underlying the graphical representations used in your figures. This is in addition to our data-deposition policy for specific types of experiments and large datasets. For readers, the source data will be made accessible directly from the figure legend. Spreadsheets can be submitted in .xls, .xlsx or .csv formats. Only one (1) file per figure is permitted: thus if there is a multi-paneled figure the source data for each panel should be clearly labeled in the csv/Excel file; alternately the data for a figure can be included in multiple, clearly labeled sheets in an Excel file. File sizes of up to 30 MB are permitted. When submitting source data files with your manuscript please select the Source Data file type and use the Title field in the File Description tab to indicate which figure the source data pertains to.

Please include a "Data availability" subsection in the Online Methods. This section should inform readers about the availability of the data used to support the conclusions of your study, including accession codes to public repositories, references to source data that may be published alongside the paper, unique identifiers such as URLs to data repository entries, or data set DOIs, and any other statement about data availability. At a minimum, you should include the following statement: "The data that support the findings of this study are available from the corresponding author upon request", describing which data is available upon request and mentioning any restrictions on availability. If DOIs are provided, please include these in the Reference list (authors, title, publisher (repository name), identifier, year). For more guidance on how to write this section please see: http://www.nature.com/authors/policies/data/data-availability-statements-data-citations.pdf

CODE AVAILABILITY
Please include a “Code Availability” subsection in the Online Methods which details how your custom code is made available. Only in rare cases (where code is not central to the main conclusions of the paper) is the statement “available upon request” allowed (and reasons should be specified).

We request that you deposit code in a DOI-minting repository such as Zenodo, Gigantum or Code Ocean and cite the DOI in the Reference list. We also request that you use code versioning and provide a license.

For more information on our code sharing policy and requirements, please see:
https://www.nature.com/nature-research/editorial-policies/reporting-standards#availability-of-computer-code

SUPPLEMENTARY PROTOCOL
To help facilitate reproducibility and uptake of your method, we ask you to prepare a step-by-step Supplementary Protocol for the method described in this paper. We encourage authors to share their step-by-step experimental protocols on a protocol sharing platform of their choice and report the protocol DOI in the reference list. Nature Portfolio's protocols.io is a free-to-use and open resource for protocols; protocols deposited onto protocols.io are citable and can be linked from the published article. More details can found at protocols.io.

ORCID
Nature Methods is committed to improving transparency in authorship. As part of our efforts in this direction, we are now requesting that all authors identified as ‘corresponding author’ on published papers create and link their Open Researcher and Contributor Identifier (ORCID) with their account on the Manuscript Tracking System (MTS), prior to acceptance. This applies to primary research papers only. ORCID helps the scientific community achieve unambiguous attribution of all scholarly contributions. You can create and link your ORCID from the home page of the MTS by clicking on ‘Modify my Springer Nature account’. For more information please visit please visit www.springernature.com/orcid.

Please do not hesitate to contact me if you have any questions or would like to discuss these revisions further. We look forward to seeing your revised manuscript and thank you for the opportunity to consider your work.

Best regards,
Madhura

Madhura Mukhopadhyay, PhD
Senior Editor
Nature Methods

Reviewers' Comments:

Reviewer #1 (Remarks to the Author):

IN their revised manuscript, Abbasi and colleagues have strongly re-organised the text and figures, and provided new experiments, adressing all the points I had raised. IN particular, they focused the entire article now on the application of their method to the extraction of 3D traction forces on confined single cells. THe results are convincing, and they better highlight the originality and specific interest of the method. They show that understanding the force response of cells requires the combination of 3D imaging, 3D traction force microscopy and a precise confinement tool with control of both spacing and stiffness of the confining surfaces. As a conclusion, I think that in terms of demonstrating the interest and novelty of the device, they now make the point more clear. THe additional applications showed in supplementary figures are also nice as an opening for futur applications.
I would suggest two small improvements:
1) the description of the results is very factual, and when reading them, it is not very clear when a finding mostly confirms prior work (like the increase in contractility induced by confinement), or when it leads to new conclusions. Also the discussion is very short and should have a chapter that specifically adresses what observation was uniquely made possible by this device, as the authors did in their point by point answer to the referees.
2) If space allows it, the discussion should also describe in more details than one global sentence, the various applications presented in supplementary figures. Even if a lot of these other applications are more preliminary, there are still very interesting observations made that could be highlighted in the discussion.

Overall, my feeling is that the device clearly opens new possibilities, and this is well demonstrated by what the authors show, but they still just scratched the surface, even for the single cell case. Maybe such a methods article is not really meant to go further than that, but it might give the impression of lack of novely, on the side of results obtained with the method. The most interesting results, in my opinion, are the single cell analysis combining forces and imaging to detect blebbing events, that

clarify greatly how the force response can be fully interpreted, as shown in the schematic proposed in figure 8 as a working model. Maybe the authors could push that a little more, and explain better in the discussion the type of wrong interpretation that could result from observing only for example the normal force, which is what has been done a lot in prior works with AFM setups.

I think these are minor points and should not represent a reason for rejecting the article.

Reviewer #2 (Remarks to the Author):

In the manuscript "CFM: Confinement Force Microscopy- a dynamic, precise, and stable microconfiner for traction force microscopy in spatial confinement" by Abbasi, Rieck, Brandt, Matis, Kiermaier, and Betz, a device is fabricated to impose parallel plate (sandwich) confinement onto both cells and cell collectives.

Confinement as a physiological microenvironmental variable is of great importance and the field's use of this variable to understand biological phenomena in both health and disease is growing rapidly. Thus, new tools that can unlock insights previously reserved for soft 2D substrates, like TFM, are in high demand. This manuscript presents a new approach for imposing this confinement upon cells, and importantly, allows for dynamic alterations of the confinement landscape, something that previous methods have lacked.

The main claims that the authors make are that they have 1) built a device that allows for real-time adjustment of vertical confinement, 2) this device allows for measurement of lateral and normal cellular forces, and 3) that this system allows them to identify a novel mechanosensitive response to confinement, namely that there is a two-step adaptation involving a stress spike and a subsequent active response that attenuates this stress, and that this active response is myosin II dependent.

Overall this is interesting research with great utility to the field, both in terms of the overall findings and in terms of the applicability of the method to a wide range of biological questions. However, there are several major issues with some of the claims, both in terms of device novelty, applicability to physiological confinement, and interpretation of the mechanotransduction-related findings.

Major points:

Relevant Literature
It is surprising, and somewhat disappointing, that after one round of revision in which one main point of feedback was that the manuscript does not properly present the current tool landscape of confinement, and specifically traction force microscopy in confinement, that some of the most important advances in this specific area are completely missing from the references, including the table showing the comparison to previous devices, which is meant to be comprehensive. One glaring omission is Afthinos/Konstantopoulos Nano Letters 2022 (PMID 36112517). This work, which was highly influential in the field's understanding of the transition point of confinement at which cells stop 'pulling' inwards and start 'pushing' outwards, found that this occurs around 4 µm x 10 µm in channel cross section- take note of Figure S4A-B, which illustrates this phenomena very clearly.

In response to previous reviewer's requests to be less biased in their assessment of their device, the authors have compiled a table to illustrate the strengths and weaknesses of each approach. However, this table is itself biased, as the only categories of assessment the authors have chosen are those that this device succeeds in. If it were to include other relevant categories, including whether the confinement geometry is physiological, their device would receive an X, as a parallel plate setup imposes only one axis of confinement (the Z axis), while the interstitial spaces present in vivo present a minimum of two axes of confinement. Thus, the Afthinos manuscript would pass this test, while the current manuscript would not. Another category of assessment that is not included is the ability to resolve traction force on both the top and bottom surfaces. This manuscript does not pass this test, while Afthinos et al would (as would Caillier/Oakes, which is discussed below).

Another point of contention that is not discussed here is the degree to which 'dynamic' loading of confinement is physiological. In comparison to the Afthinos manuscript, the authors would call this a non-dynamic system. However, this is somewhat debatable, as the dynamics of that system is directly related to the ability of the cells to dynamically migrate into the confinement, as opposed to having the confinement thrust onto them. There are certainly interesting observations that can be made in both system, and the argument can be made that neither is necessarily 'better' than the other, but the microchannel approach is likely the more physiological of the two.

Another missed citation of great importance is Caillier/Oakes JCB 2024 ("T cells use focal adhesions to pull themselves through confined environments"), which also performed TFM on single axis planar hydrogel confinement (take note of Video 7), which is very similar to the setup in this manuscript, with the notable exception of not having the ability to dynamically change the severity of confinement and the fact that this manuscript only considered lateral traction force. On the other hand, this approach was able to resolve traction forces on both the top and bottom gel, which this manuscript was unable to do.

Device Questions
In the field, the term 3D TFM is a bit of a mess, as it can refer to different experimental setups. The most basic TFM involves measuring X-Y displacements of beads on a 2D surface, which generally requires strong confocal images as the presence of out-of-plane beads that would be displaced less than surface beads would confound the resulting traction force calculations. This is uncontroversially referred to as 2D TFM. Another approach involves imaging beads below the surface, and quantifying their displacement in both X-Y and Z planes, using confocal stacks. This approach has been referred to as 3D TFM as it can

resolve 3D traction force, even though the cell that generates this force is existing on a 2D plane (some authors refer to this as 2.5 D TFM, see Delanoe-Ayari/Saw Int. J. Biochem. Cell Bio. 2023). True 3D TFM occurs in which forces are resolved around the cell in all three dimensions.

In this manuscript, 3D TFM is not performed- 2.5D TFM is, as the device can only resolve forces at the interface between the cell and a single surface. While both top and bottom surfaces have beads, all TFM calculations are done from the bottom surface only. It is understandable that the top gel might have refractive index-induced imaging artifacts. However, this greatly diminishes the overall novelty of the approach as well as the importance and usefulness of the findings. There are a number of possible confinement-induced phenomena that this setup cannot address. First, in order to fully measure lateral traction forces, the system must assume apicobasal polarity (i.e. adhesions are only present on the bottom surface). Notably, cells have been shown to alter their apicobasal and dorsovental polarity and form adhesions on their apical and dorsal sides (Wisniewski/Konstantopoulos Science Advances 2020), and thus, this system would completely miss out on the observation and calculation of this traction. The same goes for the normal stress- this system assumes the normal stress is equivalent between the top and bottom surfaces, which may be true in terms of magnitude (due to force balance requirement) but is not necessarily true in terms of distribution.

The most important experimental detail, namely how the confinement is applied, is very confusing based on what the authors have written and presented. In Figure 2, the authors show strain increasing from 0 to 60%. (Nominal strain is not defined in the methods, so we are assuming that it simply represents the displacement divided by the maximum height of the unconfined cell). Data is presented at seven levels of nominal strain- from 0 to 60%. However, the text also says that single-step confinement has been applied, which would lead one to assume that in this setup, the compression occurs in a single step, but no information is given about the total distance of this step, how long it takes to reach this step, or if imaging is done while this step is occurring (or after it has already occurred). The only information given is that the single step vertical compression is applied shortly after the 10th frame. It is unclear what 5-60% strain means- does it mean the strain goes from 5% to 60% in a single step? If so, why start at 5% and not 0%? Does it mean that there are multiple steps between 5 and 60%? Are these completely independent experiments (i.e. one set of cells is exposed to 10% strain, another to 20%, etc etc?) If so, why is there a line connecting the different strain levels, making it appear as if it is a continuous single experiment?

There is a very high amount of experimental variability in many of the results. This is especially evident in Fig. 2F, Fig. 3D and the outliers in Fig. 4C and 5C. Some of this variability appears to be papered over by not showing individual data points or curves. Given that this is a methods paper, this variability is a major cause for concern about the robustness and repeatability of the method in general. Of course, cells are known to have high levels of inherent heterogeneity, but this calls into question the choice by the authors to use transformed HeLa cancer cells that are notorious for their extreme levels of aneuploidy (they are triploid cells that have around 75 chromosomes on average). As nuclear deformation is such a major component in cellular confinement, and as HeLa nuclei have highly variable contents and morphology, this is not the appropriate model cell line to work with for this system, and likely is the culprit behind the high levels of noise in the data.

The data filtering used in Figure 6 is concerning. The authors only analyze data in which the three time points immediately after the peak had to be lower than the peak (One must assume that the peak is the increase in stress upon single step confinement, as it is not stated explicitly). The authors claim that this is because not all measured cells were exposed to a strain sufficient for reliable measurement. However, a cell not being tall enough to be exposed to sufficient strain and a cell not showing an instant decay after peak stress are not the same thing. If the filtering was that a cell must be exposed to strain over 50%, this would be more convincing. Instead, to the reader, it appears that the authors have filtered their data to only look at cells that behave the way they have predicted them to behave. In other figures the authors highlight outliers that have been left out of the measurement averages, so it’s unclear why they don’t do so here.

Minor points:
In figure 1C, the schematic appears to show that beads are only in the bottom surface, but the caption says that the beads are in both gels. The schematic should more accurately represent the setup.

In figure 2, it is unclear how the given Nominal Strain corresponds to the overall confinement distance. The authors state that this is ‘cell-size dependent’, but it’s unclear how that maps onto distance.

The authors’ statement that “once blebs began to expand, the traction forces stabilized or declined in all cells, suggesting a mechanical transition associated with morphological remodeling” is a bit presumptive, as ‘morphological remodeling’ implies an active process, and compression-induced bleb expansion is (at least mostly) a passive physical process that results from pressure exceeding the membrane’s ability to resist it.

Figure 2F is very confusing, and the data is presented in a qualitative fashion for no apparent reason. For example, what is the threshold between calling lateral force response insignificant (represented by a sideways arrow) vs calling lateral force response negative (represented by a diagonal arrow)? Is there any statistics that allow for an understanding of the generality of this response (e.g. are cells significantly more likely to downregulate their normal stress in response to compression)? Where exactly are these force responses taken from during the nearly ten minutes of step-wise compression? Wouldn’t there be differences between the first half and the second half of this process?

In figure 2D-E, putting the stress response on different axis scales does not allow the reader to fully appreciate the difference between an active cell and a dead, fixed cell.

To attribute unclear data to local microenvironment heterogeneity (4.1 line 223) is not compatible with other areas of the manuscript where the authors purport to have built a system with high levels of precision and stability (manuscript title).

Why was fibronectin-bovine serum solution used to functionalize the gels? Serum generally contains a fair amount of ECM proteins, which means there is no control over which ECM protein the cells are adhering to.

In Figure 3b, the captions says that the red line represents the ‘immediate post-confinement stress increase’. Does this also mean that the time point where this red line appears is the moment that the single-step confinement occurs? It is unclear on the x-axis where the confinement is actually applied.

In Figure 3b, what does it mean to say “Control cell”? Is this supposed to say ‘Representative cell’? Are the cells measured in Figure 3b treated differently?

This highlights a weakness in Figure 3- there are no controls or comparisons between blebbing cells and non-blebbing cells. If the non-blebbing cells have statistically similar curves, it would be inappropriate to conclude that there are major reorganizations that occur uniquely before the bleb formation.

Many statements in the results apply a fairly broad conclusion to a relatively narrow or hazy observation. For example, in the text in 4.2, the authors state that there is a transient increase in traction stress followed by stress stabilization. It is unclear what the quantitative meaning of stress stabilization is. In both the lateral and normal stress graphs in Figure 3c, there are increases in the stress after the bleb formation. If given these graphs without the annotations, it would be challenging to infer when the bleb onset occurs, and thus to simply state that stress stabilization is apparent is not a fair conclusion. The same is true for the blebbistatin section- “we did not observe […] transient stress increase prior to bleb formation followed by stress stabilization or decline” is an unquantified statement.

“In most cells we also observed that the cortical actin network becomes visibly denser prior to bleb onset, with actin accumulation evident in the preceding frames”- this statement needs to include quantified evidence in the figures.

Presenting the individual data as heatmaps is much harder to interpret than just showing 12 individual curves to illustrate the heterogeneity observed.

20 µM Y27632 is on the high side and introduces the potential for off-target effects like inhibition of PKN isoforms.

Why does the cell in Fig. 3 get compressed from 9.5 to 8 µm, while the cell in Fig. 4 gets compressed from 11 to 6 µm? Even accounting for the variety in starting height, this wouldn’t be the same level of strain. The same goes for Fig. 4, where the cell is compressed from 9 µm to 3 µm.

Given the central importance of blebbing to many of the conclusions made, it’s unclear why no rigorous quantification of blebbing, both as a function of strain and inhibitor treatment, is provided. The only information that is given is broad statements like “blebbing was largely suppressed at low to moderate strain levels (<50%), at very high strains (>60%) a subset of cells still developed blebs”. These statements are begging for accompanying graphs and statistics.

In Fig. 4C, it appears that two cells deemed outliers for average lateral stress were not deemed outliers for average normal stress (as there are four outliers on lateral and only two on normal). Given that these two quantities should be linked, it appears inappropriate to not rule out both when one is an outlier.

The formatting of the results section 4.6, with bolded highlights and arrows, is not generally appropriate for publication.

The Pearson correlation heatmap is unnecessary and should be a supplemental figure.

In the schematic in Figure 8, the nucleus has the same shape in almost every image. Is this consistent with the levels of compression applied?

The authors claim in the discussion to have achieved mechanistic clarity. Two inhibitors were used, both of which result in reduced actomyosin contractility, were used at the high end of their working concentrations. This is not the generally accepted standard for mechanistic clarity, which often requires confirmatory knockouts/knockdowns, rescue experiments, etc.

It appears that a number of experiments that have been performed for previous versions of this manuscript have been left off of this one or moved to supplemental figures. A number of experimental methods, including the use of C2C12s and CT26 cells, neutrophils, cancer spheroids, drosophila, and 9/21/114 kPa hydrogels, are included in the methods but not mentioned in the main text.

Reviewer #2 (Remarks on figshare data availability):

This is not data, it's supplementary figures.

Version 3:

Decision Letter:

Our ref: NMETH-A53559C

3rd Mar 2026

Dear Timo,

Thank you for submitting your revised manuscript "CFM: Confinement Force Microscopy-a dynamic, precise and stable microconfiner for traction force microscopy in spatial confinement" (NMETH-A53559C). It has now been seen by the original referees and their comments are below. The reviewers find that the paper has improved in revision, and therefore we'll be happy in principle to publish it in Nature Methods, pending minor revisions to satisfy the referees' final requests and to comply with our editorial and formatting guidelines.

We are now performing detailed checks on your paper and will send you a checklist detailing our editorial and formatting requirements within two weeks or so. Please do not upload the final materials and make any revisions until you receive this additional information from us.

TRANSPARENT PEER REVIEW
Nature Methods offers a transparent peer review option for new original research manuscripts submitted from 17th February 2021. We encourage increased transparency in peer review by publishing the reviewer comments, author rebuttal letters and editorial decision letters if the authors agree. Such peer review material is made available as a supplementary peer review file. **Please state in the cover letter 'I wish to participate in transparent peer review' if you want to opt in, or 'I do not wish to participate in transparent peer review' if you don't.** Failure to state your preference will result in delays in accepting your manuscript for publication.

Please note: we allow redactions to authors' rebuttal and reviewer comments in the interest of confidentiality. If you are concerned about the release of confidential data, please let us know specifically what information you would like to have removed. Please note that we cannot incorporate redactions for any other reasons. Reviewer names will be published in the peer review files if the reviewer signed the comments to authors, or if reviewers explicitly agree to release their name. For more information, please refer to our FAQ page.

ORCID
IMPORTANT: Non-corresponding authors do not have to link their ORCIDs but are encouraged to do so. Please note that it will not be possible to add/modify ORCIDs at proof. Thus, please let your co-authors know that if they wish to have their ORCID added to the paper they must follow the procedure described in the following link prior to acceptance:
https://www.springernature.com/gp/researchers/orcid/orcid-for-nature-research

Author names using non-Roman characters
Nature Portfolio journals can support presentation of author names using non-Roman characters in the HTML version of the article. If you wish to, please include author names in parentheses after the Roman-character spelling; see example online here. Currently supported scripts are: Arabic, Chinese, Cyrillic, Devanagari, Greek, Hebrew, Hangul, Japanese and Persian. You will be asked to verify the rendering is correct at proof stage.

Thank you again for your interest in Nature Methods. Please do not hesitate to contact me if you have any questions. We will be in touch again soon.

Sincerely,
Madhura

Madhura Mukhopadhyay, PhD
Senior Editor
Nature Methods

Reviewer #1 (Remarks to the Author):

In this second revision of their article, the authors have adressed the minor comments I had after their first revision, which was mostly related to the discussion part of the article. I also read in details their answer to the other reviewer, who had a very long list of comments. I think they have been all adressed satisfactorily - most of them were related to citing relevant literature, evaluation of the device and other devices for confinement, so no serious fondamental concern and it was fixed properly by the authors; other comments were more clarifications of some aspects that were correct (in my opinion) but maybe not fully clear as presented in the article; the authors have explained in details, and I think very clearly, the points of concerns and made the appropriate modifications to avoid these confusions. I think reviewer 2 really pointed to all the possible sources of misinterpretation and confusion, and the authors made a great effort to improve their article. It should now be published as it is without any need for further revisions.

Version 4:

Decision Letter:

30th Jul 2026

Dear Timo,

I am pleased to inform you that your Article, "CFM: Confinement Force Microscopy-a dynamic, precise and stable microconfiner for traction force microscopy in spatial confinement", has now been accepted for publication in Nature Methods. The received and accepted dates will be Aug 21st 2023 and Jul 30th 2026. This note is intended to let you know what to expect from us over the next month or so, and to let you know where to address any further questions.

Over the next few weeks, your paper will be copyedited to ensure that it conforms to Nature Methods style. Once your paper is typeset, you will receive an email with a link to choose the appropriate publishing options for your paper and our Author Services team will be in touch regarding any additional information that may be required. It is extremely important that you let us know now whether you will be difficult to contact over the next month. If this is the case, we ask that you send us the contact information (email, phone and fax) of someone who will be able to check the proofs and deal with any last-minute problems.

After the grant of rights is completed, you will receive a link to your electronic proof via email with a request to make any corrections within 48 hours. If, when you receive your proof, you cannot meet this deadline, please inform us at rjsproduction@springernature.com immediately.

Authors may need to take specific actions to achieve compliance with funder and institutional open access mandates. If your research is supported by a funder that requires immediate open access (e.g. according to Plan S principles or the NIH public access policy) then you should select the gold OA route, and we will direct you to the compliant route where possible. Because authors warrant under our subscription licensing terms that they haven’t committed to licensing any version of their article under a licence inconsistent with the terms of our agreement – including the applicable embargo period – publication under the subscription model isn’t suitable for authors whose funders require no embargo.

If you have any questions about our publishing options, costs, Open Access requirements, or our legal forms, please contact ASJournals@springernature.com

If you have posted a preprint on any preprint server, please ensure that the preprint details are updated with a publication reference, including the DOI and a URL to the published version of the article on the journal website.

You may wish to make your media relations office aware of your accepted publication, in case they consider it appropriate to organize some internal or external publicity. Once your paper has been scheduled you will receive an email confirming the publication details. This is normally 3-4 working days in advance of publication. If you need additional notice of the date and time of publication, please let the production team know when you receive the proof of your article to ensure there is sufficient time to coordinate. Further information on our embargo policies can be found here: https://www.nature.com/authors/policies/embargo.html

To assist our authors in disseminating their research to the broader community, our SharedIt initiative provides you with a unique shareable link that will allow anyone (with or without a subscription) to read the published article. Recipients of the link with a subscription will also be able to download and print the PDF.

As soon as your article is published, you will receive an automated email with your shareable link.

If you are active on Twitter/X or Bluesky, please e-mail me your and your coauthors’ handles so that we may tag you when the paper is published.

You can now use a single sign-on for all your accounts, view the status of all your manuscript submissions and reviews, access usage statistics for your published articles and download a record of your refereeing activity for the Nature journals.

Please note that you and any of your coauthors will be able to order reprints and single copies of the issue containing your article through Nature Portfolio's reprint website, which is located at http://www.nature.com/reprints/author-reprints.html. If there are any questions about reprints please send an email to author-reprints@nature.com and someone will assist you.

Please feel free to contact me if you have questions about any of these points.

Best regards,
Madhura

Madhura Mukhopadhyay, PhD
Team Leader and Senior Editor
Nature Methods

** Visit the Springer Nature Editorial and Publishing website at www.springernature.com/editorial-and-publishing-jobs for more information about our career opportunities. If you have any questions please click here.**

Open Access This Peer Review File is licensed under a Creative Commons Attribution 4.0 International License, which permits use, sharing, adaptation, distribution and reproduction in any medium or format, as long as you give appropriate credit to the original author(s) and the source, provide a link to the Creative Commons license, and indicate if changes were made. In cases where reviewers are anonymous, credit should be given to 'Anonymous Referee' and the source.
The images or other third party material in this Peer Review File are included in the article's Creative Commons license, unless indicated otherwise in a credit line to the material. If material is not included in the article's Creative Commons license and your intended use is not permitted by statutory regulation or exceeds the permitted use, you will need to obtain permission directly from the copyright holder.
To view a copy of this license, visit https://creativecommons.org/licenses/by/4.0/

Response to Reviewers

Manuscript: *CFM: Confinement Force Microscopy-a dynamic, precise and stable microconfiner for traction force microscopy in spatial confinement*

Authors: Abbasi *et al.*

Manuscript ID: **NMETH-A53559A-Z**

Dear editor, dear reviewers,

We thank you for taking the time to read our manuscript and to provide such constructive and thoughtful feedback. We revised the manuscript to be more balanced in tone, focused on the advantages the *dual-axis* (lateral & normal) provides to get new insights on the biological systems studied under dynamic confinement, and we now emphasize in greater detail the scope and limitations of the CFM. Below we respond point-by-point to the comments provided by the reviewers in the previous session. Due to the major changes, we keep the rebuttal letter short and focus on answering the questions regarding parts that have remained in the main text of the manuscript.

To summarize the changes:

1. **Introduction rewritten** for balanced tone; overstatements removed; comparative claims moved to **Supplementary Table S1**.
2. **Core focus** on single-step confinement in HeLa with **dual-axis insights** (diverging trends; coupling vs. stress relief; timing vs. blebbing).
3. **Contractility perturbations clarified:** Y-27632 reduces stresses strikingly and abolishes blebs; blebbistatin lowers stresses yet permits high-strain blebs without pre-bleb transients.
4. **Oscillation, embryo, spheroid** moved to Supplementary as capability vignettes.
5. **Methods/notation** clarified (dual-axis TFM choice; viscoelastic mapping; statistics).
6. **Literature context expanded** (Raman 2013; Hui 2019; Brunner 2006; Ofek 2009 and related), with neutral, feature-based comparisons.

Reviewer #1

Remarks to the Author: *In this article, Abbasi and colleagues propose a novel confiner device that allows a precise control of the confinement height combined with simultaneous measurement of lateral and normal forces (3D traction force). The strength of the device is the combination of these three features, as they were all achieved previously independently of each other, or in combination of two of them, but not the three. The device is well characterized and rather simple. Most of the article is dedicated to short proofs of principle for applications of the method to a variety of contexts, from single cells to developing embryos or cell aggregates. This makes it clear that the device will be useful for a large set of researchers. The drawback is that none of the application is really pushed far enough to convincingly make the point of the unique capacity of this method to bring novel insights. While the method is a clear technical breakthrough, the article remains, in its present form, too superficial in the applications it proposes for the method.*

Response: We thank the reviewer for the encouraging comments. We followed the suggestions of the reviewers and changed to focus on a single application as described in the point by point response below.

Main concerns:

Comment 1- Writing: *The current style of the article is, being a bit caricatural, a little like an advertisement for a new product, combined with some application notes. I think this should be changed. In the introduction, listing the previous confinement devices is useful, but the way their drawbacks is presented against all the advantages of the new method is not so interesting and often a bit biased if not almost incorrect (like when the authors say that 'the new method simulate the in vivo mechanical and chemical environments experienced by cells and tissue' - this is a clear overstatement!). One can propose a table for such comparisons, but for the intro, it is more interesting to present what has been achieved so far and the important discoveries made with confinement devices, to show that this is an interesting topic for cell biology, and then simply present the novel possibilities brought by the present device, and it is self-evident that these new implementations were not possible with previous devices. The focus being obviously the combination of confinement with a 3D force measure and what can be done with this combination.*

Response: We thank the reviewer for the direct criticism. To improve the paper, we rewrote the Introduction almost completely to now emphasize biological questions that can be adressed with the CFM and to better acknowledge prior discoveries. We also now describe previous tools in more balanced terms, and focused on the advantages of our CFM without overselling by saying now: "Unlike previous approaches, the CFM permits time-resolved confinement while preserving full imaging access and allows downstream analysis of cellular force responses from the acquired stress maps." (Lines 52-54)

Furthermore, Supplementary Table S1 now provides a neutral, feature-based comparison.

Comment 2. *The choice of the authors to show many possible applications is at the expense of going far enough in any of them. I think it is not a very good choice. Maybe the authors could remove some (or just mention them in a sup figure) and spend more time on showing specifically what new can be learned from the combination of confinement with 3D force measures. In particular, I think that what needs to be shown is that knowing both the normal and lateral stress makes a difference in the understanding of the cell response. For example, relaxation can be well studied with just the normal or just the lateral forces, especially if they are similar in both axis (is it the case?). Similarly, the change in the force exerted by the cells as the confinement increases, and for various confinement levels, can be studied independently for the normal force (with an AFM, as previously published by several teams), or only laterally with regular 2D traction force. So the authors should show an application when measuring forces on both axis on the same cell makes a difference.*

Response: We thank the reviewer for guiding us to a stronger manuscript. For the revised version, we follow the advice of the reviewer and focus now on a single-step confinement assay in HeLa cells and show how *dual-axis* readouts change interpretation. This was done by performing new experiments and providing additional analysis approaches, such as analyzing forces, and blebbing behavior and correlating forces, confinement responses and shape changes at the cell level. In detail we demonstrate:

- In about half of the cells measured, the traction forces after a confinement step in the **lateral and normal directions develop differently** (Fig. 2f), revealing mechanical decoupling of these two force components that would be missed if only the lateral or only the normal forces are measured, as currently done in other approaches.

- To answer the reviewer's question regarding the independence of the lateral and normal stress, we provide a focused view on this aspect in the revised version. We find that only in about half of the cells investigated, the lateral and normal stress show the same trend after a step confinement. To better quantify this, we used the Pearson correlation value between the normal and lateral stress, which gives a moderate correlation (r=0.40) that confirms that about half of the cells show a similar trend. In a systematic comparison between all quantities measured, we can now show that in cells where normal and lateral stress behavior are more strongly correlated, we also find a more pronounced release of the normal stress after the confinement step (correlation between normal-lateral coupling and the decrease in normal stress after confinement: $r = -0.56$; Fig. 7). Thus, lateral–normal coupling is characteristic of pressure relief during mechanical adaptation, whereas in other phases the components may co-increase or transiently decouple or diverge.

- A **transient dual-axis stress enhancement** commonly precedes the onset of bleb formation in cells that had not already shown blebbing in the initial confinement. Once

the bleb expansion starts, the lateral and normal stresses either stabilize or even decline (Fig. 3a–d).

To improve the paper, we have included these findings in the Results section and Figures 3–8.

Comment 3: *Taking specifically each proposed application, there are a few obvious points that the authors should address to substantiate the results that they report:*

Comment 3a. *On the confinement of single HeLa cells and neutrophiles (figures 2 and 3): several reports have shown the induction of persistent blebbing upon confinement, or at least a higher contracility (in immune cells). Did the authors also observe that? For example, is the plateau in the stress increase for the very low confinement, on neutrophiles, due to blebbing, which could reduce the stress? And how much of the stress observed is due to the cell cortex contractility (comparing with cells treated with Y27632 for example)? It is also not clear whether the stress increase is an active response (in neutrophiles), or rather the absence of stress increase (in HeLa). As the authors show, a passive object shows an increase in stress as it gets deformed. Is it what is observed on neutrophils before the plateau for the very low confinement? Or do they increase the stress more, suggesting an increase in contractility, as reported for other cells (including immune cells) before? And for Hela cells, is the absence of increase due to blebbing? Others, using an AFM device, have reported an increase in stress, more similar to the 'passive' response, until the cell eventually increases contractility, leading to blebbing. Could the difference also be due to the adhesive versus non adhesive state of cells (neutrophiles, or the Hela studied in previous studies, are not adhesive or on non adhesive substrates, while here cells are adhesive, especially the HeLa cells). Can the authors modulate the adhesiveness of the substrate? This is a lot of points to address, but a complete study on one aspect is more interesting, even for a methods paper, than a lot of rather superficially studied examples*

Response: We agree with the reviewer that indeed such questions regarding the effect of myosin contractility versus blebbing are important. In the revised manuscript we include new data and provide additional data analysis. In detail, we now report blebbing frequencies/timing as a function of stress. In control cells (untreated with Y-27632), a transient stress rise typically precedes bleb onset, but then stabilizes or even declines with ongoing expansion (Fig. 3). In contrast, **Y-27632** treatment reduces stresses markedly, even after a step confinement, and abolishes blebbing (Fig. 4). This is consistent with previous reports that an *actomyosin-dependent* active component is key for blebbing in confinement. Furthermore, **blebbistatin** lowers stresses after a step confinement, yet still permits bleb formation in very high-strain ($> 60\%$) situations (Fig. 5), consistent with passive rupture of the actomyosin cortex under extreme compression.
To improve the paper, we have added all of these finding to the results section and Figs. 3–5.

Comment 3b. *The durotaxis example is really great and appears also more novel and more unique to the possibilities offered by the presented method, because of the two gels. One aspect in which it could be completed is to also report the inverse situation, in which cells are attached initially to a soft roof and then transit to a stiffer bottom gel, which would allow to report the forces on the stiff substrate towards which cells migrate, and complement what the authors report on the soft gel side, that the cells leave. In addition, it would be useful to document the state of focal adhesion during the process, to correlate with the forces. Finally, a discussion of how these results compare to what was reported on forces that cells exert when spreading between two plates (for example from the Asnacios lab) would be interesting.*

Response: We thank the reviewer for the suggestion. In the revised manuscript, we implemented the requested *soft-roof* → *stiff-bottom* configuration (top 1.2 kPa → bottom 114 kPa; Supplementary Video S11) and confirmed robust transitions. However, we could not *quantify* tractions on the stiff bottom gel: neutrophils are weakly adhesive and transition rapidly (sometimes < 3 min), producing no resolvable bead displacement in 114 kPa gels; consequently, the TFM inversion returned no stress signal. Lowering the "stiff" substrate to $\sim$50 kPa increased sensitivity but abolished the fast durotaxis within our 30 min observation window. We therefore report the configuration that yields measurable forces (soft bottom → stiff top) and state this limitation explicitly.

To visualize the focal adhesions, we attempted viral and transient expression of *paxillin*, *vinculin*, and *talin* in the neutrophils used (both in the Betz and Kiermaier labs), but *transfection was unsuccessful*, so no usable FA labeling was obtained. Fixation under confinement was not possible without disrupting the cells. Accordingly, we do not present FA analyses for neutrophils in this assay.

We recognize that a detailed comparison to "spreading between two plates" is of interest. However, we decided to follow the reviewer's suggestion to focus on a few situations; therefore, we did not include a dedicated subsection in this revision and instead focused on datasets where dual-axis readouts provide the clearest added value (single-step HeLa assay).

Comment 3c. *What is reported for the oscillating constraint appears very preliminary. It is just the feasability, and even that is not really investigated (how fast can it be done while measuring forces for example?). The interpretation of the result is very qualitative for such a quantitative output. Maybe, for this aspect, just studying in more details one dynamic confinement step would be more interesting (relaxation, etc...) and give the opportunity to focus on what the XYZ force measure brings: for example is relaxation the same in both axis?*

Response: As requested by the reviewer, in the revised manuscript we now refocused on single-step dynamics in the main text. As the oscillation data is still interesting, we decided to move oscillation to the Supplementary Material to demonstrate further possible instrument capabilities. Normal-axis relaxation fits are now included in the main text of the

manuscript and are reported (Figure 6a)

Comment 3d. *The application on the drosophila embryo is even less detailed and is just showing that the embryo survives the growth in the device. This is a bit anecdotical.*

Response: These results are now fully moved to the Supplementary Information and streamlined. We prefer to include them, simply to demonstrate such embryos can also be used with the instrument.

Comment 3e. *Spheroid: This part is interesting but it is not clear at all in which sense it is specific to this device. It seems that here only the normal stress is exploited, which means that this could be done with any classical method (and it has been done indeed).*

Response: We also decided to move this part to the Supplementary Information to demonstrate the CFM's capability to measure properties of larger tissue models.

Reviewer #2

Remarks to the Author:

A. Key results: *The authors report confinement force microscopy. This method allows to confine cells, spheroids, organoids, embryos into micrometer spacings while at the same time measuring traction forces. It can be combined with fluorescence microscopy, so that also biological changes can be recorded.*

B. Originality: *The method is not intrinsically novel. It is certainly a more precise version compared to previous approaches. However, having in mind the following references, the general strategy of the approach does not appear novel enough for me to justify publication in Nature methods. For example, the following references, which also combined confinement with (traction) force measurements, have not been mentioned in the manuscript:*

*• Raman et al., Probing cell traction forces in confined microenvironments (2013):
https://pubs.rsc.org/en/content/articlelanding/2013/LC/c3lc50802a*

*• Hui et al., Cell traction force in a confined microenvironment with double-sided micropost arrays (2019):
https://pubs.rsc.org/en/content/articlelanding/2019/RA/C8RA10170A*

*• Brunner et al., Cell migration through small gaps (2006):
https://link.springer.com/article/10.1007/s00249-006-0079-1*

In addition, also cytocompression is not new and the simple combination with traction force

microscopy is a nice and biophysically interesting aspect to study, but again in my opinion this does not justify publication in Nature Methods:

• There are several reports on cytocompression assays, including Ofek et al., 2009: https://www.cell.com/fulltext/S0006-3495(09)01301-0

***C.** Data and methodology: The data are very well prepared, the methodology is an improved version of previously published similar approaches. The quality of the data and of the presentation is excellent and the device is more precise than others before, but the innovation is limited compared the previous ones.*

D. Statistics: Appropriate.

E. Conclusions: They are robust, but I am missing a discussion in the context of the above mentioned literature.

F. Suggestions: I do not see that the approach is novel enough for publication in Nature Methods. It is a great paper, and an improvement of previous approaches, but not original enough.

G. References: See missing references above.

H. Clarity: Appropriate.

Response: We appreciate the positive assessment of data quality and presentation. We agree the previously published papers contain related approaches, but we would like to point out that these do not allow these capabilities to be combined in a single experimental approach. Hence, the contribution here is not higher precision alone but the integration of capabilities that, to our knowledge, have not been available previously in one platform:

1. **Real-time, closed-loop tunable vertical confinement** with sub- μm drift over 10–20 h;
2. **Simultaneous, spatially resolved lateral *and* normal stresses** on *continuous* compliant substrates during live imaging;
3. **Time-resolved** measurements aligned to the confinement step and to **morphological events** (e.g., bleb onset) within the same cell;
4. Compatibility with high-NA objectives and tiled fields for improved **throughput**.
5. The ability to trigger and capture **rapid** cell responses (e.g., neutrophil durotaxis, prebleb transients) under well-defined, two-surface stiffness contrasts.

This combination enables biological inferences that would be *missed or ambiguous* with single-axis or fixed-gap setups (e.g., post-step trend decoupling between lateral and normal stress, cross-axis coupling vs. normal-stress relief, and perturbation-specific trajectories). To make this positioning explicit, we (1) added a concise comparison table, contrasting CFM

with prior confiners/TFM variants, highlighting which features were previously separate or only available in pairs; (2) clarified the analysis pipeline and (3) tempered wording around novelty while emphasizing the new biological readouts made possible by the combined design. We also expanded the limitations note (e.g., quantification on very stiff substrates) to give a balanced view.
We also cite and discuss the mentioned previous studies in the Introduction and Supplementary Table S1.
We hope that now the added value of the CFM becomes sufficiently clear. We think that all these features together are very important, but have not been accessible in one instrument before.

Response to Reviewers

Manuscript: *CFM: Confinement Force Microscopy-a dynamic, precise and stable microconfiner for traction force microscopy in spatial confinement*

Authors: Abbasi *et al.*

Manuscript ID: **NMETH-A53559B**

Reviewer #1

Remarks to the Author:

In their revised manuscript, Abbasi and colleagues have strongly re-organised the text and figures, and provided new experiments, adressing all the points I had raised. In particular, they focused the entire article now on the application of their method to the extraction of 3D traction forces on confined single cells. The results are convincing, and they better highlight the originality and specific interest of the method. They show that understanding the force response of cells requires the combination of 3D imaging, 3D traction force microscopy and a precise confinement tool with control of both spacing and stiffness of the confining surfaces. As a conclusion, I think that in terms of demonstrating the interest and novelty of the device, they now make the point more clear. The additional applications showed in supplementary figures are also nice as an opening for future applications. I would suggest two small improvements: 1) the description of the results is very factual, and when reading them, it is not very clear when a finding mostly confirms prior work (like the increase in contractility induced by confinement), or when it leads to new conclusions. Also the discussion is very short and should have a chapter that specifically adresses what observation was uniquely made possible by this device, as the authors did in their point by point answer to the referees. 2) If space allows it, the discussion should also describe in more details than one global sentence, the various applications presented in supplementary figures. Even if a lot of these other applications are more preliminary, there are still very interesting observations made that could be highlighted in the discussion.

Overall, my feeling is that the device clearly opens new possibilities, and this is well demonstrated by what the authors show, but they still just scratched the surface, even for the single cell case. Maybe such a methods article is not really meant to go further than that, but it might give the impression of lack of novely, on the side of results obtained with the method. The most interesting results, in my opinion, are the single cell analysis combining forces and imaging to detect blebbing events, that clarify greatly how the force response can be fully interpreted, as shown in the schematic proposed in figure 8 as a working model. Maybe the

authors could push that a little more, and explain better in the discussion the type of wrong interpretation that could result from observing only for example the normal force, which is what has been done a lot in prior works with AFM setups.

I think these are minor points and should not represent a reason for rejecting the article.

Response: We sincerely thank the reviewer for the very positive evaluation of our revised manuscript and for acknowledging the substantial reorganization, new experiments, and clearer focus on 3D traction-force extraction under confinement. We are grateful for the reviewer's recognition that the method's novelty and value are now clearly demonstrated, and we appreciate the constructive suggestions below. We have implemented all recommendations as detailed here.

1. Clarifying which findings confirm prior work versus provide new insights, and expanding the discussion

We fully agree with the reviewer. In response, we have substantially changed and expanded the *Discussion* (lines 550–752) and revised it to clearly separate results that primarily confirm prior work from those that provide new mechanistic insight enabled by CFM.

First, we now explicitly state where our findings are in line with previous work, both in the *Results* and in the *Discussion*. In particular, in the *Discussion* paragraph beginning with "Aligning traction stresses to the timing of bleb onset…" (lines 573–587), we relate the confinement-induced increase in cortical contractility and intracellular pressure directly to earlier studies [Liu *et al.*, *Cell*, 2015; Lomakin *et al.*, *Science*, 2020; Yang *et al.*, *Biophysical Journal*, 2021]. We also added a corresponding statement in the *Results* to ensure that this connection to prior work is visible at the point where the phenomenon is first presented (lines 302–305). We then explain how CFM extends these models by providing access to the full, time-resolved force dynamics around bleb onset in individual cells and by identifying a stereotyped sequence of stress increase, bleb expansion, and stress relaxation.

Second, we now emphasize in the *Discussion* several **new insights that are uniquely enabled by CFM**, including:

- the decoupling of bleb initiation from myosin II–dependent force generation under extreme confinement, revealed by blebbistatin experiments where robust blebbing occurs with minimal stress and no clear stress rise at bleb onset (lines 648-655);
- the simultaneous extraction of single-cell viscoelastic parameters and force dynamics from the same normal-stress relaxation curves, showing that contractility inhibition softens cells and thereby reduces the passive stress spike (lines 606-618);
- the distinction between normal and lateral stress components during adaptation, demonstrating active redistribution of forces between vertical and in-plane directions that would be invisible to normal-force-only approaches (lines 656-668);

Finally, we added a dedicated paragraph (beginning with "A unifying theme of these findings..." on lines 696-707) that explicitly summarizes the **unique capabilities of CFM as a device**: dynamically programmable confinement, simultaneous lateral and normal traction readout, and access to viscoelastic parameters at the single-cell level. We explain how this combination enables observations such as the bleb-associated stress time series, the contractility-independent blebbing under extreme confinement, and the coordinated redistribution of forces, thereby directly addressing the reviewer's request for a section that highlights what is uniquely made possible by CFM.

2. Expanding the discussion of additional applications shown in the Supplementary Figures

We thank the reviewer for this suggestion. As recommended, we have expanded the *Discussion* to describe the Supplementary applications in more detail (lines 732-752). Specifically, we now briefly summarize each use case illustrated in Supplementary Figures S1–S5. These additional examples are now highlighted the *Discussion* to further demonstrate the breadth and future potential of CFM beyond the main HeLa-cell studies focused on in the paper.

3. Emphasizing bleb–force coupling and explaining risks of misinterpretation when only normal forces are measured

We greatly appreciate the reviewer's emphasis on the bleb–force coupling as a particularly interesting aspect of the work, and we have strengthened the *Discussion* accordingly. In particular, we now explicitly discuss the types of misinterpretation that can arise when only normal forces are measured, as is common in AFM-based compression assays (lines 619-668).

In the paragraph beginning "A central advantage of CFM over approaches that report only normal forces..." (lines 619-636), we explain how measurements of both normal and lateral stresses show that the immediate response to a confinement step can involve an increase in normal stress while lateral stress decreases. We point out that, if only normal forces were measured, such responses could be interpreted as a uniform change in the cell's mechanical state, whereas the combined readout reveals active redistribution of forces between vertical and in-plane directions.

In the subsequent paragraph, beginning "These advantages become particularly clear in the context of blebbing..." (lines 637-655), we now explicitly address the reviewer's point. We show that, in untreated cells, both normal and lateral stresses rise around bleb onset, supporting the working model summarized in Fig. 8, where increased cortical contractility and pressure precede bleb expansion and subsequent stress relaxation. We then contrast this with blebbistatin-treated cells, where pronounced blebbing occurs under strong confinement with very low stresses and no clear stress rise at bleb onset in either component. We explain that a normal-force-only measurement might suggest modest contractility changes associated with blebs, whereas the combined lateral and normal signals make it clear that, under these

conditions, blebs can arise with minimal traction stress generation, pointing to contractility-independent pathways of bleb initiation under extreme confinement.

Together, these additions clarify how CFM prevents misinterpretation of confined-cell mechanics that could arise when only normal forces are available and directly connect the experimental findings to the working model in Fig. 8.

Reviewer #2

Remarks to the Author:

In the manuscript "CFM: Confinement Force Microscopy- a dynamic, precise, and stable microconfiner for traction force microscopy in spatial confinement" by Abbasi, Rieck, Brandt, Matis, Kiermaier, and Betz, a device is fabricated to impose parallel plate (sandwich) confinement onto both cells and cell collectives.

Confinement as a physiological microenvironmental variable is of great importance and the field's use of this variable to understand biological phenomena in both health and disease is growing rapidly. Thus, new tools that can unlock insights previously reserved for soft 2D substrates, like TFM, are in high demand. This manuscript presents a new approach for imposing this confinement upon cells, and importantly, allows for dynamic alterations of the confinement landscape, something that previous methods have lacked.

The main claims that the authors make are that they have 1) built a device that allows for real-time adjustment of vertical confinement, 2) this device allows for measurement of lateral and normal cellular forces, and 3) that this system allows them to identify a novel mechanosensitive response to confinement, namely that there is a two-step adaptation involving a stress spike and a subsequent active response that attenuates this stress, and that this active response is myosin II dependent.

Overall this is interesting research with great utility to the field, both in terms of the overall findings and in terms of the applicability of the method to a wide range of biological questions. However, there are several major issues with some of the claims, both in terms of device novelty, applicability to physiological confinement, and interpretation of the mechanotransduction-related findings.

Response: We thank the reviewer for the overall positive evaluation of our approach, and the constructive criticism that we reply to below in a point by point response, and which helped us to greatly improve the manuscript.

Major points:

Relevant Literature. It is surprising, and somewhat disappointing, that after one round of revision in which one main point of feedback was that the manuscript does not properly present the current tool landscape of confinement, and specifically traction force microscopy

in confinement, that some of the most important advances in this specific area are completely missing from the references, including the table showing the comparison to previous devices, which is meant to be comprehensive. One glaring omission is Afthinos/Konstantopoulos Nano Letters 2022 (PMID 36112517). This work, which was highly influential in the field's understanding of the transition point of confinement at which cells stop 'pulling' inwards and start 'pushing' outwards, found that this occurs around 4 μ m $\times$ 10 μ m in channel cross section- take note of Figure S4A-B, which illustrates this phenomena very clearly.

Response: We thank the reviewer for this important remark and acknowledge and apologize that our previous revision did not yet adequately reflect the existing landscape of confinement-based traction force microscopy. In particular, we agree that the work by Afthinos and Konstantopoulos (Nano Letters, 2022; PMID 36112517) is a key contribution that should have been explicitly cited.

In the revised manuscript, we have now:

- Added explicit citations to Afthinos *et al.* (Konstantopoulos lab) in both the Introduction and Supplementary Table 1, highlighting it as a key example of confinement in well-defined compliant microchannel geometries—with precisely controlled dimensions/topography (line 41)—that is augmented by direct traction-force measurements during migration (Supplementary Table 1).
- Clarified in the *Discussion* how our parallel-plate CFM geometry is conceptually complementary to such microchannel-based approaches: whereas Afthinos and Konstantopoulos probe confinement in 2D cross-sectional channels with traction readout on confining walls, our device focuses on dynamically tunable plate spacing with simultaneous lateral and normal traction readout at one interface, enabling single-cell viscoelastic analysis and controlled confinement histories (lines 708-725).

Reviewer's comment: *In response to previous reviewer's requests to be less biased in their assessment of their device, the authors have compiled a table to illustrate the strengths and weaknesses of each approach. However, this table is itself biased, as the only categories of assessment the authors have chosen are those that this device succeeds in. If it were to include other relevant categories, including whether the confinement geometry is physiological, their device would receive an X, as a parallel plate setup imposes only one axis of confinement (the Z axis), while the interstitial spaces present in vivo present a minimum of two axes of confinement. Thus, the Afthinos manuscript would pass this test, while the current manuscript would not. Another category of assessment that is not included is the ability to resolve traction force on both the top and bottom surfaces. This manuscript does not pass this test, while Afthinos et al would (as would Caillier/Oakes, which is discussed below).*

Response: We thank the reviewer for this important critique and agree that, in its previous form, the comparison table could be perceived as biased, because it primarily listed capabili-

ties in which CFM performs well. Our intention was to highlight the specific mechanobiological features that motivated the device design (dynamic confinement control and simultaneous lateral/normal readout at one interface), but we fully agree that omitting categories where other tools clearly excel does not provide a balanced overview. In this regard, we have implemented the following changes:

(i) Revising the comparison table and adding missing evaluation criteria.
In the revised manuscript, we have updated the comparison table (Supplementary Table 1) to include additional criteria that the reviewer rightly points out as important for assessing physiological relevance:

- **Confinement geometry (which directions are constrained)** (e.g. 1D plate-like apico–basal compression in a parallel-plate setup; 2D cross-sectional confinement in microchannels that restrict both width and height; or full 3D confinement within fibrous matrices that constrain the cell in all directions).
- **Typical in vivo situations best represented by each geometry** (e.g. apico–basal compression of epithelial or endothelial layers, cell transit through pore- or capillary-like constrictions, or mesenchymal migration in interstitial ECM).

Rather than assigning a single scalar judgement of "more" or "less" physiological, we followed the reviewer's advice to focus on relevant categories and in the revised table we now specify for each method the class of biological scenarios for which its geometry is most appropriate. For example, parallel-plate confinement as implemented in CFM and AFM is annotated as being particularly suited to processes dominated by apico–basal compression against an extended substrate (such as crowded epithelial or endothelial monolayers, cells beneath stiff capsules or bone, or compressed tissue slabs and organoids), whereas microchannel systems such as Afthinos/Konstantopoulos are annotated as especially relevant for cell transit through pore- or capillary-like constrictions (e.g. leukocyte extravasation or cancer cell passage through narrow vessels). Likewise, 3D fibrous matrices are indicated as best capturing situations in which cells are embedded in and constrained by a deformable fiber network, as in mesenchymal migration through collagen-rich stroma or endothelial invasion into ECM.

(ii) Why we do not treat dual-surface traction readout as a simple comparison category.
We fully agree with the reviewer that, conceptually, resolving traction forces on both confining surfaces would be highly desirable. We would like to stress that this is not a limitation we ignored: during method development we systematically cross-checked the physical possibility to access the TFM on the top gel and already mentioned the associated issues in Section 2.2. We realize that our explanation there was too condensed, and we now expand it more clearly in both the *Methods* (section 5.7, lines 920-931) and *Discussion* (lines 669-682).

In brief, the refraction generated by the cell them-self generates a detectable image distor-

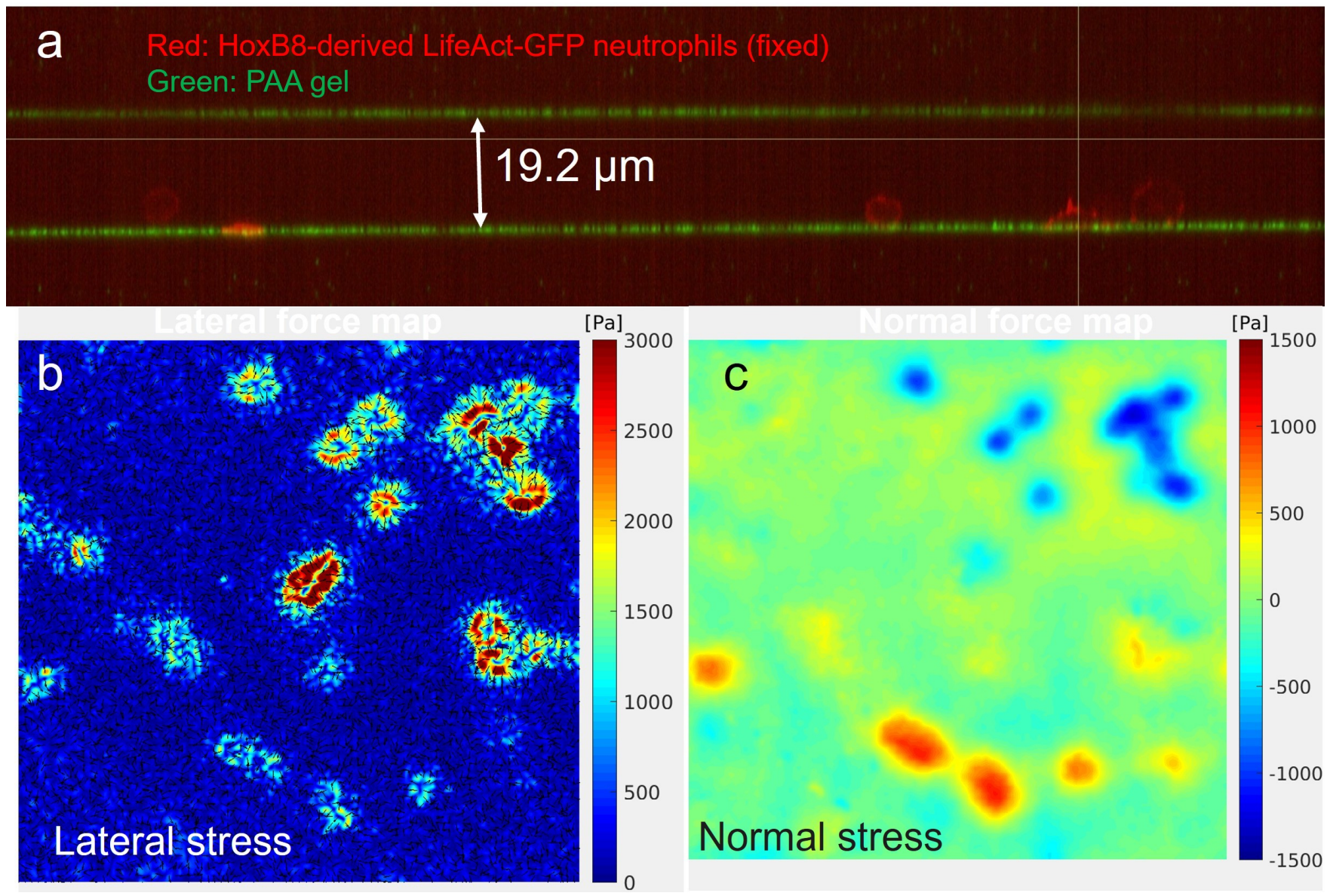

Figure 1: **Apparent top-gel traction stresses in the absence of cell–gel contact reveal refractive-index artefacts.** **(a)** Side-view fluorescence image of Hoxb8-derived neutrophils expressing LifeAct-GFP (red) seeded on the bottom PAA gel, which is positioned at a distance of 19.2 μ m from the bottom gel. Green signal shows fluorescent beads embedded in the PAA gels. At this spacing, the cell does not physically contact the top gel at all. **(b)** Lateral stress map reconstructed from bead displacements in the top gel for the position shown in (a). The inferred lateral stresses reach values up to $\sim$ 3000 Pa, i.e. more than 20-fold higher than the typical lateral tractions generated by neutrophils (mean $\sim$ 150 Pa; Supplementary Fig. S1). **(c)** Normal stress map for the same position, showing apparent normal stresses up to $\sim$ 1500 Pa, again $\sim$ 20-fold above the usual neutrophil normal stresses (mean $\sim$ 70–80 Pa; Supplementary Fig. S1). Because these unrealistically large stresses are obtained when the cell is far from the top gel and therefore cannot transmit mechanical forces to it, they indicate that top-gel traction reconstructions are dominated by refractive-index-induced imaging artefacts rather than true cell-generated forces.

tion, which results in force artifacts that leads to wrong recognition of pulling forces even in situation where the confinement should lead to cells pushing on the walls. We demonstrated this effect by imaging beads in the top gel and running the traction stress calculations, where we observed substantial apparent "stress" on the top surface even when the top gel was positioned far above the cell (e.g. $\sim$ 15 μm away, with no physical contact). In these control experiments, the reconstructed top-gel stress could even exceed the values obtained when the gel was brought into contact ($\sim$ 20-fold above the usual neutrophil traction stresses), indicating that these signals were artefactual (Figure 1 in the Rebuttal letter). This behaviour is consistent with the known problems arising from refractive-index mismatch between the cell and the surrounding medium, which distorts bead positions and leads to false traction signals. The problem is general for all microscopy methods that image through a cell, and is well described in literature by Stephan et al (Nano Lett. 2017, 17, 12, 8018–8023).

To further confirm the effect, we tested refractive-index matching of the medium to the cell using OptiPrep. As predicted by the cell refraction artifact explanation, this substantially reduced the optical errors, but it introduced a different and equally problematic effect: the increased medium density caused weakly adhesive cells to float and also weakened integrin-mediated adhesion in more adhesive cell types. Under these conditions, the measured tractions no longer reflect the native mechanics of cells in standard medium, undermining the physiological relevance of the assay.

We also explored a computational correction strategy by modifying our TFM code for the top gel to account for distortions as a function of cell shape and local density. However, this would require cell-specific corrections that depend on heterogeneous, time-varying parameters such as organelle positioning, local cell thickness, and internal density variations—not only between cells but even within a single cell over time. In practice, we could not identify a robust correction scheme that would yield reliable absolute traction values at the top interface.

Finally, as in the quasi-static conditions of our experiments, the cell has negligible acceleration in the z-direction, so Newton's second law implies

$$\sum F_z \;=\; F_{\text{bottom}} + F_{\text{top}} + F_{\text{grav}} \;\approx\; 0.$$

The gravitational term $F_{\text{grav}} = mg$ is extremely small compared with the nanonewton traction forces we measure, and buoyancy forces are similarly negligible. Thus, to an excellent approximation, the normal forces transmitted to the top and bottom gels must balance:

$$F_{\text{top}} \;\approx\; -F_{\text{bottom}}.$$

This final check furthermore demonstrated that the measured traction forces on the top are not consistent with simple physical laws.

Based on these experimental and mechanical considerations, we decided to adopt a conservative approach: we restrict quantitative traction-force analysis to the bottom gel, where bead imaging and inversion are robust and well controlled, and we use force-balance arguments to interpret the net normal load. In the revised manuscript, we now (i) state this explicitly in Section 2.2 (lines 181-190), and (ii) mention in the *Discussion* that dual-surface traction readout remains an important technical goal for the field but is presently limited by refractive-index artefacts and medium-induced changes in cell adhesion and mechanics (lines 669-682). For this reason, we do not present "dual-surface traction" as a simple check-box category in the comparison table, but instead discuss it explicitly in the text as a current limitation of our implementation.

We would also like to point out that we suspect that this error is also affecting the results from Afthinos/Konstantopoulos and Caillier/Oakes, as the presented forces in both papers

suggest that the sum of forces is not 0 as required by Newton's laws. Furthermore, both papers do not address the problem of optical refraction or any technical method that might have used to overcome this quite fundamental limitation.

We hope that these results can convince the reviewer that the top and bottom TFM measurements are not a good category for the assessment of the different methods, as there remain questions regarding the effect of the optical refraction generated by the cells.

Reviewer's comment: *Another point of contention that is not discussed here is the degree to which 'dynamic' loading of confinement is physiological. In comparison to the Afthinos manuscript, the authors would call this a non-dynamic system. However, this is somewhat debatable, as the dynamics of that system is directly related to the ability of the cells to dynamically migrate into the confinement, as opposed to having the confinement thrust onto them. There are certainly interesting observations that can be made in both system, and the argument can be made that neither is necessarily 'better' than the other, but the microchannel approach is likely the more physiological of the two.*

Response: We thank the reviewer for raising this important conceptual point. We agree that the term "dynamic loading" can describe different biological situations depending on whether confinement arises because the cell *moves into* a constriction (as in microchannel systems) or because the *microenvironment compresses* the cell (as in externally imposed plate compression). These represent distinct but complementary physiological scenarios. Microchannel-based systems such as Afthinos/Konstantopoulos indeed model situations in which cells actively migrate into a constriction—for example leukocyte entry into capillaries or pore-like structures. In this special context, the spatial dynamics of confinement are cell-driven, and the rate of confinement is dictated by the migration speed. We fully agree that for studying squeezing-driven migration through pores or channels, this geometry is particularly physiological.

However, many *in vivo* situations involve externally imposed, time-varying compression rather than cell-driven entry into confinement. Examples include tissue-level compression during development, crowding in epithelia, compression by stromal cells or tumour mass, pressure fluctuations in dense immune microenvironments, or mechanically loaded tissues. In these settings, confinement is not self-generated by migration but arises from forces applied to the cell from outside, often at time scales that exceed or bypass the cell's ability to regulate the loading rate. CFM is designed precisely to model such externally imposed temporal changes in confinement by offering programmable single-step or cyclic changes in height with micrometer precision. Thus, we agree with the reviewer that neither approach should be considered inherently "better" or universally "more physiological"; rather, each mimics a different class of biological constraints. To improve the manuscript, we now clarify this explicitly in the *Discussion* (lines 708-725). Parallel-plate confinement captures situations dominated by apico–basal compression, whereas microchannel-based confinement captures scenarios dominated by geometrical constriction during migration. In this sense, CFM and

microchannels are complementary tools that address different mechanobiological questions.

Reviewer's comment: *Another missed citation of great importance is Caillier/Oakes JCB 2024 ("T cells use focal adhesions to pull themselves through confined environments"), which also performed TFM on single axis planar hydrogel confinement (take note of Video 7), which is very similar to the setup in this manuscript, with the notable exception of not having the ability to dynamically change the severity of confinement and the fact that this manuscript only considered lateral traction force. On the other hand, this approach was able to resolve traction forces on both the top and bottom gel, which this manuscript was unable to do.*

Response: We thank the reviewer for pointing us to the study by Caillier and Oakes (*J. Cell Biol.*, 2024), which we now cite explicitly in the revised manuscript. Their work is an important contribution to confinement-based traction force microscopy and, as the reviewer notes, shares conceptual similarities with our planar hydrogel configuration.

As detailed in our response to the previous point and in the revised *Results* and *Discussion* (Section 2.2 and *Discussion*), extensive control experiments during CFM development led us to adopt a conservative strategy regarding dual-surface traction analysis. In brief, we found that under standard imaging conditions refractive-index–induced distortions produce apparent tractions in the upper gel even when it is positioned far above the cell, and that index-matching media, while reducing these artefacts, substantially alter cell adhesion and mechanics. One hallmark of these artifacts are resulting (wrong) traction forces that are not pointing towards the cell center, but towards the edge of the cells. Such wrong force detection is easily explained by the refraction of the cell, but is very difficult to explain by intracellular processes (actin filaments can pull, but buckle under pushing forces). On this basis, we concluded that we cannot obtain quantitatively reliable traction maps from the top gel without compromising physiological relevance, and we therefore restrict all quantitative traction analysis in CFM to the bottom gel in the present study.

In light of these observations, we treat dual-surface traction reconstruction as an important technical goal for future development, rather than as a criterion we can robustly satisfy at this stage. If other studies managed to overcome the problems remains open, as the optical effect was not discussed in these papers, and no clear controls have been shown demonstrating that the optical artifact is irrelevant for them. For this reason, we do not use top-gel traction as a benchmarking category in the comparison table, and we refrain from evaluating specific quantitative interpretations of studies that report such measurements. We therefore cite the Caillier/Oakes study as a relevant example of planar confinement TFM (line 42), while focusing our own analysis on the aspects of traction measurement that can be performed reliably within the constraints of our optical and mechanical system.

Reviewer's comment: Device Questions

In the field, the term 3D TFM is a bit of a mess, as it can refer to different experimental se-

tups. The most basic TFM involves measuring X-Y displacements of beads on a 2D surface, which generally requires strong confocal images as the presence of out-of-plane beads that would be displaced less than surface beads would confound the resulting traction force calculations. This is uncontroversially referred to as 2D TFM. Another approach involves imaging beads below the surface, and quantifying their displacement in both X-Y and Z planes, using confocal stacks. This approach has been referred to as 3D TFM as it can resolve 3D traction force, even though the cell that generates this force is existing on a 2D plane (some authors refer to this as 2.5 D TFM, see Delanoe-Ayari/Saw Int. J. Biochem. Cell Bio. 2023). True 3D TFM occurs in which forces are resolved around the cell in all three dimensions.

Response: We thank the reviewer for this useful clarification regarding terminology in the traction force microscopy field. We agree that the terms "2D TFM", "2.5D TFM", and "3D TFM" are used inconsistently across the literature, with different groups emphasizing either the dimensionality of the *cell–substrate interface* or the dimensionality of the *reconstructed force vector field*. As the reviewer notes, several authors refer to approaches that reconstruct the full (F_x, F_y, F_z) force vector on a single planar substrate as "2.5D TFM," since the cell adheres to a geometrically 2D surface even though out-of-plane forces can be computed from 3D bead displacements.

In the interest of consistency with this nomenclature, and to avoid implying that we perform near-cell–encapsulating volumetric force reconstructions (which would constitute "true 3D TFM" in the strict sense), we now explicitly describe our approach as a *3-component (2.5D) traction stress reconstruction* (line 159). This terminology reflects both perspectives: (i) we reconstruct all three components of the traction stress vector, including the normal component, using 3D bead displacements, and (ii) these forces are resolved on a planar elastic substrate rather than within a volumetric matrix.

We have adjusted the wording in the manuscript accordingly (line 159) and added a brief explanation in the revised *Methods* (section 5.7; lines 914-918) to clarify that CFM measures the full vectorial stress components at a single interface but does not attempt volumetric stress reconstruction around the entire cell body. This terminology more accurately situates our work within the existing landscape while preserving the mathematical correctness of the force reconstruction performed.

Reviewer's comment: *In this manuscript, 3D TFM is not performed- 2.5D TFM is, as the device can only resolve forces at the interface between the cell and a single surface. While both top and bottom surfaces have beads, all TFM calculations are done from the bottom surface only. It is understandable that the top gel might have refractive index-induced imaging artifacts. However, this greatly diminishes the overall novelty of the approach as well as the importance and usefulness of the findings. There are a number of possible confinement-induced phenomena that this setup cannot address. First, in order to fully measure lateral traction forces, the system must assume apicobasal polarity (i.e. adhesions are only present on the bottom surface). Notably, cells have been shown to alter their apicobasal and dorsoventral polarity and form adhesions on their apical and dorsal sides (Wisniewski/Konstantopoulos*

Science Advances 2020), and thus, this system would completely miss out on the observation and calculation of this traction. The same goes for the normal stress- this system assumes the normal stress is equivalent between the top and bottom surfaces, which may be true in terms of magnitude (due to force balance requirement) but is not necessarily true in terms of distribution.

Response: We thank the reviewer for this thoughtful comment and for highlighting an important conceptual distinction between the dimensionality of the traction readout and the dimensionality of confinement. As the reviewer mentioned correctly in this comment and we described in our responses to the previous points, and now stated explicitly in the *Methods* and *Discussion*, extensive control experiments demonstrated that reliable dual-surface traction reconstruction is physically not feasible under standard imaging conditions due to refractive-index–induced artefacts in the upper gel. For this reason, we adopt a deliberately conservative strategy and restrict quantitative traction analysis to the bottom gel only. We therefore do not base any claims of device novelty on dual-surface TFM, nor do we interpret traction patterns on the top gel. Importantly, the central novelty of CFM does not rely on measuring tractions on both confining surfaces. Rather, the advance introduced here is the combination of (i) *dynamically programmable* confinement with micrometre precision, applied during live imaging; (ii) the *simultaneous* reconstruction of lateral and normal traction-stress components at a single interface; and (iii) the extraction of single-cell viscoelastic parameters from the same experiment.

This integrated capability allows us to obtain time-resolved force dynamics during externally imposed confinement transitions—something not achievable with previous parallel-plate systems or conventional 2D TFM—and to uncover mechanistic features such as the stereotyped force sequence around bleb onset and the decoupling of bleb initiation from myosin II–dependent force production.

Regarding apicobasal polarity, we agree with the reviewer that some cell types can form adhesions on their dorsal or apical surfaces under certain conditions (e.g. Wisniewski/Konstantopoulos, *Science Advances*, 2020). Our measurements do not assume that such adhesions are absent; rather, they reflect the well-controlled traction field at the bottom interface, which is the only interface for which quantitative reconstruction is optically reliable. Even if apical adhesions contribute to force transmission, as acknowledge by the reviewer, Newton's third law ensures that net normal forces on the top and bottom gels must balance in the quasi-static regime relevant for our experiments. As the reviewer correctly mentioned in this comment and we discussed in our answer to the previous comments, what may differ between the two surfaces, is the *spatial distribution* of forces, which is indeed an interesting biological question. If users wish to measure apical traction forces, cells can be cultured on the top gel (in an inverted configuration). In this setup, the bottom gel contacts the apical side of the cells, enabling traction measurements from the apical interface.

Regarding adhesions, the reviewer refers to integrin-mediated cell–ECM attachment sites (focal adhesions or related adhesive contacts) that transmit traction to a substrate. We agree

that under confinement some cells can form adhesive contacts on the upper (apical/dorsal) surface (e.g. Wisniewski/Konstantopoulos, Science Advances, 2020), and CFM does not *assume* that such adhesions are absent: the upper gel can be ECM-coated and cells may adhere to it in some configurations, as we observed in our durotaxis experiments. The limitation is instead optical/technical: under standard imaging conditions top-gel bead displacements cannot be reconstructed reliably, and therefore any traction field inferred for the top interface would be quantitatively incorrect. For this reason, the stresses reported throughout the manuscript should be interpreted explicitly as *bottom-interface* traction stresses. We have clarified this point in the revised *Results/Methods/Discussion* and added it as a limitation and future direction, namely that robust dual-surface traction reconstruction would be required to quantify apical traction distributions under confinement.
For these reasons, we respectfully contend that the inability to reconstruct top-gel tractions does not diminish the novelty or usefulness of CFM. The method was not designed to probe dorsal traction patterns, but to enable externally imposed confinement with real-time mechanical readout of both normal and lateral stresses, together with viscoelastic characterization—an experimental capability that is unique among existing confinement tools and central to the mechanistic insights presented in this work.

Reviewer's comment: *The most important experimental detail, namely how the confinement is applied, is very confusing based on what the authors have written and presented. In Figure 2, the authors show strain increasing from 0 to 60%. (Nominal strain is not defined in the methods, so we are assuming that it simply represents the displacement divided by the maximum height of the unconfined cell). Data is presented at seven levels of nominal strain-from 0 to 60%. However, the text also says that single-step confinement has been applied, which would lead one to assume that in this setup, the compression occurs in a single step, but no information is given about the total distance of this step, how long it takes to reach this step, or if imaging is done while this step is occurring (or after it has already occurred). The only information given is that the single step vertical compression is applied shortly after the 10th frame. It is unclear what 5-60% strain means- does it mean the strain goes from 5% to 60% in a single step? If so, why start at 5% and not 0%? Does it mean that there are multiple steps between 5 and 60%? Are these completely independent experiments (i.e. one set of cells is exposed to 10% strain, another to 20%, etc etc?) If so, why is there a line connecting the different strain levels, making it appear as if it is a continuous single experiment?*

Response: We thank the reviewer for pointing out that our description of the confinement protocols was not sufficiently clear. We understand that, in its previous form, the manuscript could give the impression that the multi-step experiment in Fig. 2d-e and the single-step experiments in Figs. 3–5 represent the same type of loading protocol. They are in fact two distinct experimental paradigms, performed with different goals and different imaging strategies. We have now revised the text to make this explicit.

(i) Clarification of Fig. 2d-e (multi-step confinement). In Fig. 2d-e we applied *three consecutive confinement steps* to each HeLa cell and measured the traction response at each step. The goal of this experiment was purely to determine whether traction stress scales systematically with increasing confinement. Here, the "nominal strain" corresponds to the change in cell height divided by the cell height before the *first* confinement step (line 208). Because HeLa cells are highly heterogeneous in size, the same absolute gel–gel distance results in different initial compression levels across cells. Reporting strain relative to each cell's initial height therefore allows the confinement level to be compared meaningfully across the dataset. It also means that some cells may already experience mild pre-confinement before the first step, which is an unavoidable consequence of size variability and is precisely why strain normalization is essential. The line segments connecting the points in Fig. 2d are shown only to visualize the trend with strain and do not indicate a continuous time series from the same cell. We now explain this clearly in the *Results* (lines 212-214).

(ii) Clarification of the single-step confinement experiments (Figs. 3–5) and Fig. 2f. The remainder of the manuscript (Figs. 3–5 and downstream analyses) is based on a completely separate experimental design: a *single, rapid confinement step* applied during high-temporal-resolution dual-channel imaging (cell + gel). Here, we explicitly attempted to begin each experiment with the cell minimally confined—i.e. the gels positioned such that the cell is just touching the top substrate without appreciable compression. Because of cell-to-cell size heterogeneity, a small degree of initial confinement is unavoidable in some cells, but in this dataset the fluorescence signal of the cell is acquired, allowing us to determine the cell size and the blebbing phase of each cell at the onset of confinement.

Fig. 2f summarizes *the same single-step confinement paradigm* (a single rapid confinement step applied during a ∼8.5 min recording, with stresses analyzed over the full time series), but it was acquired in an *earlier implementation* of the setup in which only the **gel channel** was recorded continuously. This was a practical limitation of the camera used at the time: acquiring the full 3D stacks required for traction reconstruction at 5 s resolution did not permit simultaneous dual-channel (cell+gel) volumetric imaging over 100 frames. Consequently, for the dataset used in Fig. 2f we recorded the gel stacks for traction analysis throughout the experiment, while the **cell channel was captured only as snapshots immediately before and immediately after** the 100-frame gel time series.

For this reason, Fig. 2f is intentionally a *categorical summary* of the long-term traction trends (increase/decrease/no change) across a large population, whereas the mechanistic analyses in Figs. 3–5 rely on a later dual-channel (cell+gel) implementation that enables time-resolved alignment of traction dynamics with bleb initiation and cell-shape changes. We have now stated this explicitly in the Fig. 2f caption to avoid the impression that Fig. 2f includes continuous morphology readout. In addition, we performed a quantitative correlation analysis (Fig. S6) using Fig. 2f and Fig. 3-5 showing that pre-confinement systematically modulates both stress amplitudes and the timing of bleb onset. Thus, pre-confinement is not an unobserved nuisance variable but an explicit part of the dataset: because we record

the fluorescence of the cell and the bleb dynamics, we can interpret each stress time series in the context of the cell's initial confinement state and blebbing phase, rather than assuming that all cells start from an identical mechanical condition.

In the single-step protocol, imaging begins with 10 time-points of 3D stack acquisition in an unconfined state (or partially pre-confined). The confinement step is applied *immediately after the 10th Time-point* and occurs within less than one second, i.e. between stacks 10 and 11, and is therefore not captured as an intermediate image stack. This allows us to align traction dynamics precisely to the onset of confinement.

We thank the reviewer again for highlighting this issue; the revised text now clearly delineates the two experimental paradigms (lines 204-221), defines nominal strain (line 208), explains why strain normalization is required (lines 209-212), and clarifies the temporal structure of the single-step loading protocol (lines 230-244).

Reviewer's comment: *There is a very high amount of experimental variability in many of the results. This is especially evident in Fig. 2F, Fig. 3D and the outliers in Fig. 4C and 5C. Some of this variability appears to be papered over by not showing individual data points or curves. Given that this is a methods paper, this variability is a major cause for concern about the robustness and repeatability of the method in general. Of course, cells are known to have high levels of inherent heterogeneity, but this calls into question the choice by the authors to use transformed HeLa cancer cells that are notorious for their extreme levels of aneuploidy (they are triploid cells that have around 75 chromosomes on average). As nuclear deformation is such a major component in cellular confinement, and as HeLa nuclei have highly variable contents and morphology, this is not the appropriate model cell line to work with for this system, and likely is the culprit behind the high levels of noise in the data.*

Response: We thank the reviewer for this thoughtful comment. We fully agree that the variability in the stress measurements is high, particularly at stronger confinement, and we appreciate the opportunity to clarify its origin. Importantly, this variability does not arise from instability or lack of robustness of the CFM device, but from well-understood sources of biological and geometric heterogeneity.

(i) Origin of variability at the single-cell level. The dominant contributor to variability is the large heterogeneity in HeLa cell size, cell height, and nuclear morphology. Because strain is defined relative to each cell's initial height, even small differences in starting geometry translate into substantially different absolute deformations at higher nominal strain. Under strong confinement, this geometric amplification leads to diverging stress magnitudes even when cells follow similar mechanical behaviour. This is also why fixed cells show increased standard deviation at 50–60% strain (Fig. 2e): at these high compressions the response becomes dominated by geometric constraints and nonlinear strain-stiffening, so small differences in initial cell height/shape and contact area translate into disproportionately different stresses. In addition, living HeLa cells differ in adhesion distribution, cortical tension, and

pre-confinement state, all of which contribute to heterogeneous stress evolution. The correlation analysis introduced in the revised manuscript (Fig. S6) explicitly demonstrates that part of this variation is biologically meaningful and systematically relates to differences in pre-confinement level.

Beyond differences in stress *amplitude*, an important component of the spread in Fig. 3D and Fig. 4f arises because cells are captured at different *phases* along a common adaptation pathway to confinement. Our time-resolved single-step experiments show that most cells exhibit a stereotyped sequence—an early passive stress spike, an active stress buildup, and a stabilization/relaxation phase that often coincides with bleb expansion—but the timing of these transitions varies across cells. Consequently, population snapshots at a fixed time point naturally appear broad: cells that have not yet entered the contraction/blebbing phase, cells currently undergoing active buildup, and cells already in the post-bleb relaxation phase can coexist within the same condition. This contribution is not related to the choice of HeLa cells.

(ii) On the use of HeLa cells. We agree that HeLa cells are heterogeneous. This was, in fact, one of the reasons for selecting them as an initial test system: their variability provides an accurate benchmark for whether a method can access time-resolved force changes reliably at the single-cell level. The key observations reported here—including the force peak preceding bleb onset, the relaxation associated with bleb expansion, and the decoupling of bleb initiation from myosin-II–dependent forces under extreme confinement—are not inferred from pooled averages. They arise from individual time series, where the relevant transitions are directly observed within each cell. Thus, the biological heterogeneity of HeLa cells does not obscure the mechanistic conclusions. Additionally, the effect of blebs as function of confinement has been largely studied in HeLa cells. To benchmark our method with respect to the knowledge in the field, the advantages of HeLa cells outweigh in our opinion the difficulties mentioned by the reviewer.

(iii) Transparency of data presentation. To address the reviewer's concern, we have clarified in the text and figure captions that the spread in the data reflects biological and geometric sources of variation rather than instrumental noise (lines 276-284). We have also added concise explanations (e.g. in the caption of Fig. 2e) describing why variability increases at strong confinement and how this relates to differences in initial height and nuclear deformation.

(iv) Robustness of the CFM method. Despite the biological variability in HeLa cells, the CFM platform yields stable, reproducible stress time series at high temporal resolution. The device's precision is demonstrated by the smoothness of individual stress curves, the consistency of viscoelastic parameters extracted from relaxation fits, and the ability to resolve coordinated dynamics of normal and lateral stress components. The observed variability is therefore a property of the biological system, not a limitation of the instrument.

In summary, while HeLa cells exhibit substantial heterogeneity—particularly under strong

compression—this variability is expected, biologically interpretable, and explicitly accounted for in our analysis. It does not compromise the robustness of the CFM method nor the mechanistic conclusions derived from single-cell trajectories.

Reviewer's comment: *The data filtering used in Figure 6 is concerning. The authors only analyze data in which the three time points immediately after the peak had to be lower than the peak (One must assume that the peak is the increase in stress upon single step confinement, as it is not stated explicitly). The authors claim that this is because not all measured cells were exposed to a strain sufficient for reliable measurement. However, a cell not being tall enough to be exposed to sufficient strain and a cell not showing an instant decay after peak stress are not the same thing. If the filtering was that a cell must be exposed to strain over 50%, this would be more convincing. Instead, to the reader, it appears that the authors have filtered their data to only look at cells that behave the way they have predicted them to behave. In other figures the authors highlight outliers that have been left out of the measurement averages, so it's unclear why they don't do so here.*

Response: We thank the reviewer for raising this point; it highlights an important limitation of the relaxation analysis in Fig. 6. Thanks to the reviewer, we now extend our explanation regarding the filtering criterion, and discuss biases the dataset toward cells whose relaxation dynamics are slow enough to be temporally resolved (lines 353-366).

(i) Why a peak-based filter is necessary at the current temporal resolution. In our single-step experiments, one full 3D stack of the gel is acquired approximately every 5 s (i.e. $\sim$0.2 Hz). Several cells relax much faster than this, with most of their decay occurring within one or two frames after the confinement step. For such cells, the stress peak and the majority of the relaxation occur between sampling points, making any exponential fit mathematically meaningless (due to overfitting) and dominated by temporal undersampling rather than actual mechanical behaviour.

For this reason, we required that the three time points immediately following the force peak have to be lower than the peak itself. This criterion does not enforce a particular biological behaviour; it ensures only that enough data points exist to support a valid relaxation fit at our sampling rate. We fully acknowledge that this excludes the fastest-relaxing cells from the relaxation analysis.

(ii) Consequence: Fig. 6 characterizes only the mechanically resolvable subset. Because fast relaxation typically corresponds to lower effective stiffness and/or viscosity in standard viscoelastic models, the subset that passes the filter consists of cells whose relaxation dynamics are slow enough to be captured at 5 s intervals. Fig. 6 therefore does not describe the full population of confined cells but rather the mechanically resolvable subset. However, because our inclusion criterion preferentially excludes the fastest, low-amplitude relaxations (which are more frequent under myosin II inhibition), it cannot artificially create a softening effect; if anything, it makes our reported E_0 conservative. Therefore, the true population-level

softening under blebbistatin is likely at least as strong as what we report, and our conclusion that myosin II inhibition markedly reduces stiffness (E_0)—with a trend toward lower viscosity—remains unchanged. We now state this clearly in the revised manuscript (lines 353-366) and Fig. 6 caption, and we have softened the wording in the main text accordingly.

We emphasize that this limitation affects *only* the viscoelastic parameter extraction. Our main conclusions on confinement-induced stress peaks, blebbing dynamics, and drug effects (Figs. 3–5, 7) are based on the complete datasets and are not filtered in this way.

(iii) Why a strain-based threshold would not solve the issue. We agree that insufficient strain can hinder peak detection, but high strain does not ensure that relaxation is resolvable, as the relaxation rate is a cell property. Some highly compressed cells relax extremely quickly and cannot be sampled adequately at 5 s intervals, while other moderately compressed cells relax more slowly and yield clean peaks. A strain-only threshold (e.g. >50%) would therefore exclude many analyzable cells and include many traces that cannot be reliably fitted. A dynamics-based criterion is therefore more appropriate for identifying traces suitable for mechanical fitting.

(iv) Outlook: fast-relaxing cells could be included with faster volumetric imaging. In principle, if one could acquire 3D stacks over the required field of view at much higher frame rates (sampling intervals well below the characteristic relaxation time), then even the fastest-relaxing cells could be analyzed without filtering. At present, however, achieving such volumetric acquisition speeds is beyond the capabilities of our camera and scanning system, which must cover a significant z-range at each time point. Thus, we chose a conservative but transparent approach: we report viscoelastic parameters only where the data support a meaningful fit and explicitly acknowledge the limitation.

Reviewer's comment: Minor points: *In figure 1C, the schematic appears to show that beads are only in the bottom surface, but the caption says that the beads are in both gels. The schematic should more accurately represent the setup.*

Response: We thank the reviewer for pointing this out. The schematic in Fig. 1C has now been corrected to accurately depict fluorescent beads in both the top and bottom gels.

Reviewer's comment: *In figure 2, it is unclear how the given Nominal Strain corresponds to the overall confinement distance. The authors state that this is 'cell-size dependent', but it's unclear how that maps onto distance.*

Response: We thank the reviewer for this helpful comment and agree that our original description of "nominal strain" was not sufficiently clear. In Fig. 2, the nominal strain is calculated on a per-cell basis as

$$\varepsilon_{\text{nominal}} = \frac{h_0 - h_{\text{confined}}}{h_0},$$

where h_0 is the height of the cell immediately before the first confinement step and h_{confined} is the height after a given step. Because HeLa cells exhibit substantial heterogeneity in initial height, the same absolute gel-to-gel distance can correspond to different strains for different cells. For this reason, strain does not map to a single fixed confinement distance but instead reflects each cell's relative deformation with respect to its own starting height.

The intention of Fig. 2 was therefore not to encode the absolute plate spacing, but to illustrate how traction stresses scale with increasing *cell-internal* deformation across cells of different sizes. We have clarified this definition explicitly in the revised *Results* (lines 207-209) and improved the wording in the Fig. 2 caption.

Reviewer's comment: *The authors' statement that "once blebs began to expand, the traction forces stabilized or declined in all cells, suggesting a mechanical transition associated with morphological remodeling" is a bit presumptive, as 'morphological remodeling' implies an active process, and compression-induced bleb expansion is (at least mostly) a passive physical process that results from pressure exceeding the membrane's ability to resist it.*

Response: We thank the reviewer for pointing out the distinction between the mechanically passive inflation of a bleb and the upstream and downstream cellular processes that regulate it. We agree that the outward expansion of a bleb is driven by intracellular pressure overcoming local membrane–cortex attachment, which is a largely passive physical process. Our statement, however, refers not to the inflation step itself, but to the transition in force dynamics that accompanies bleb initiation.

In our experiments, the increase in traction stress prior to bleb onset, and its subsequent stabilization or decline, are strongly dependent on actomyosin contractility and are abolished or altered under ROCK inhibition or myosin II inhibition. This indicates that the pressure buildup preceding bleb formation, and the force redistribution that follows it, are *actively regulated* rather than purely passive. To avoid ambiguity, we have revised the text to clarify that the "mechanical transition associated with morphological remodeling" refers to these active processes—cortical contractility changes, pressure regulation, and adhesion adjustment—while the inflation of the bleb itself remains a passive mechanical consequence (lines 254-258).

Reviewer's comment: *Figure 2F is very confusing, and the data is presented in a qualitative fashion for no apparent reason. For example, what is the threshold between calling lateral force response insignificant (represented by a sideways arrow) vs calling lateral force response negative (represented by a diagonal arrow)? Is there any statistics that allow for an understanding of the generality of this response (e.g. are cells significantly more likely to downregulate their normal stress in response to compression)? Where exactly are these force responses taken from during the nearly ten minutes of step-wise compression? Wouldn't there be differences between the first half and the second half of this process?*

Response: We thank the reviewer for these comments and agree that the rationale behind

Fig. 2F was not sufficiently explained in the previous version. We clarify below how this panel was generated, what the arrows represent, and why the analysis is qualitative.

(i) What Fig. 2F represents. Fig. 2F summarizes the *single-step confinement experiments* in which each HeLa cell was imaged for 100 time points (5 s per time point; total duration ~8.5 min) and a single confinement step was applied between time points 10 and 11. For every cell we therefore obtain a complete stress time series over the entire experiment. Fig. 2F is a compact, categorical representation of the *overall trend* (increase, decrease, or no change) in lateral and normal stress over this 8.5-minute period. It is not intended as a quantitative display of the full time series (which are shown in detail in Figs. 3–5), but as a visual summary of how normal and lateral stresses jointly evolve during adaptation.

(ii) How categories and arrows were defined. In the revised caption of Fig. 2f we now state explicitly how the trends were determined. For each cell, we compared the stress level in an early post-step window with the stress level in a late window near the end of the recording. To ensure that these values reflect the stabilized part of the signal, the last 15 frames were analyzed using a 7-frame moving-average smoothing, and a linear fit was used to quantify the tail slope. A cell was considered stable if (i) the magnitude of the tail slope did not exceed three times the median absolute deviation of the temporal derivative in the same window, and (ii) the standard deviation did not exceed 0.8 times the interquartile range. Cells passing these criteria were used to determine whether the late stress was higher, lower, or unchanged relative to the early post-step window.

Arrows in Fig. 2F encode this joint trend classification: upward (↑) = increased normal stress; downward (↓) = decreased normal stress; sideways (↔) = no substantial change. We now make this more clear in the figure caption.

(iii) Where in the time series the values are taken. The reviewer asks whether the analysis is based on the first or second half of the 8.5-minute recording. The quantities used for Fig. 2F compare (1) an early post-step window immediately after the confinement step with (2) a late stabilized window derived from the last 15 frames. This allows us to capture the *net adaptation* over the entire experiment rather than an instantaneous value at a single time point.

(iv) On qualitative representation and generality. Although Fig. 2F is qualitative by design, we also report in the figure the fraction of cells in each category (increase, decrease, no change) so that readers can assess how frequently each response occurs. This provides a quantitative sense of generality without turning Fig. 2F into a second, redundant version of the detailed time-resolved analyses presented in Figs. 3–5, which constitute the main mechanistic basis of the study.

We hope that these clarifications make the purpose, construction, and interpretation of Fig. 2F fully transparent.

Reviewer's comment: *In figure 2D-E, putting the stress response on different axis scales does not allow the reader to fully appreciate the difference between an active cell and a dead, fixed cell.*

Response: We thank the reviewer for this helpful observation. The intent of Fig. 2d-e was to show the *shape* of the stress dynamics rather than their absolute magnitudes, which differ by more than an order of magnitude between live and fixed cells. Because fixed cells generate normal and lateral stresses that are extremely small compared to active cells, plotting both traces on the same axis would compress the fixed-cell curve to the point of being visually indiscernible. For this reason, separate axes were used so that both behaviors remain readable.

To address the reviewer's concern, we have now clarified this explicitly in the caption. This preserves interpretability while avoiding a figure in which the fixed-cell data would be effectively invisible.

Reviewer's comment: *To attribute unclear data to local microenvironment heterogeneity (4.1 line 223) is not compatible with other areas of the manuscript where the authors purport to have built a system with high levels of precision and stability (manuscript title).*

Response: We thank the reviewer for raising this point. The intention of the sentence was not to suggest that the CFM instrument lacks mechanical stability or precision. Rather, the term "local microenvironmental heterogeneity" was meant to refer to biological and biochemical factors that vary at the cell–substrate interface and are well known to influence traction-force readouts in all TFM-based approaches (e.g. spatially heterogeneous adhesion density, local variations in cortex–substrate coupling, or cell-generated remodeling of the ECM coating). These sources of heterogeneity arise even when the physical confinement geometry is imposed with high precision, as in our system, and therefore do not contradict the mechanical stability of the CFM device.

To avoid misunderstanding, we have revised this sentence in the manuscript to make explicit that the heterogeneity we refer to is *biological* rather than instrumental (lines 276-288). The revised text now clarifies that CFM provides stable and uniform confinement, and that the observed variability in stress trajectories reflects cell-intrinsic differences (e.g. cell size, nuclear mechanics, cytoskeletal organization) rather than any technical imprecision.

Reviewer's comment: *Why was fibronectin-bovine serum solution used to functionalize the gels? Serum generally contains a fair amount of ECM proteins, which means there is no control over which ECM protein the cells are adhering to.*

Response: We thank the reviewer for raising this point and apologize for the confusion caused by our previous wording. We did not use fetal bovine serum (FBS) for gel functionalization. In all experiments, fibronectin was diluted in PBS and used as a defined coating.

The misunderstanding stems from an imprecise phrasing in an earlier version of the *Meth-*

ods, where we wrote "fibronectin–bovine serum." This was incorrect. What we intended to indicate was fibronectin from bovine plasma (i.e., the source of the purified fibronectin), not a serum-containing coating mixture. We have now revised the *Methods* accordingly to explicitly state the coating procedure (fibronectin in PBS; lines 778-779).

Reviewer's comment: *In Figure 3b, the captions says that the red line represents the 'immediate post-confinement stress increase'. Does this also mean that the time point where this red line appears is the moment that the single-step confinement occurs? It is unclear on the x-axis where the confinement is actually applied.*

Response: We thank the reviewer for pointing out this ambiguity. Yes—the red line marks the interval in which the single-step confinement is applied. In our protocol, confinement is triggered after time point $T10$ (i.e. 45 s after the start of imaging, given a 5 s frame interval), and the red line spans from $T10$ to the next acquired time point $T11$ (i.e. from 45 s to 50 s after the start of imaging). Thus, the beginning of the red line indicates the moment the confinement step is initiated, and its end corresponds to the first post-step image time point. We have now indicated $T11$=50s directly on the figure and clarified this in the caption as well as the *Results* (lines 233-236).

Reviewer's comment: *In Figure 3b, what does it mean to say "Control cell"? Is this supposed to say 'Representative cell'? Are the cells measured in Figure 3b treated differently? This highlights a weakness in Figure 3- there are no controls or comparisons between blebbing cells and non-blebbing cells. If the non-blebbing cells have statistically similar curves, it would be inappropriate to conclude that there are major reorganizations that occur uniquely before the bleb formation.*

Response: We thank the reviewer for this important point. In Figure 3b, "control" denotes vehicle control: cells were treated with the same (maximal) DMSO concentration used in the inhibitor conditions to ensure comparability across treatments (Figs. 3,4,5).

We agree that, in principle, comparing cells that undergo blebbing to cells that do not bleb would be valuable to assess whether the observed pre-onset stress dynamics are specifically associated with bleb initiation rather than reflecting a generic time-dependent response to confinement.

In our single-step confinement experiments, however, blebbing is highly prevalent: in more than 90% of cells we observe blebs within the recording window (or cells are already blebbing at the start of imaging). As a consequence, the number of cells without an identifiable bleb onset during the acquisition is small, and these cases are likely influenced by the finite observation window rather than representing a stable "non-blebbing" phenotype.

Importantly, the analysis in Fig. 3 is explicitly restricted to cells for which a bleb onset time can be identified, because aligning stress time series to bleb onset requires this reference point. We have now clarified this explicitly in the *Results* (lines 291-294) and in the Fig. 3

caption to avoid any implication that Fig. 3 represents a comparison between blebbing and non-blebbing populations.

Consistent with the reviewer's concern, we do not generalize the onset-aligned features to all confined cells. Instead, we now state more precisely that the characteristic stress increase and subsequent stabilization/decline shown in Fig. 3 is observed in cells that undergo bleb initiation during the recording, and we avoid wording that would imply this behaviour is necessarily present in cells without detectable blebbing.

Reviewer's comment: *Many statements in the results apply a fairly broad conclusion to a relatively narrow or hazy observation. For example, in the text in 4.2, the authors state that there is a transient increase in traction stress followed by stress stabilization. It is unclear what the quantitative meaning of stress stabilization is. In both the lateral and normal stress graphs in Figure 3c, there are increases in the stress after the bleb formation. If given these graphs without the annotations, it would be challenging to infer when the bleb onset occurs, and thus to simply state that stress stabilization is apparent is not a fair conclusion. The same is true for the blebbistatin section- "we did not observe [...] transient stress increase prior to bleb formation followed by stress stabilization or decline" is an unquantified statement.*

Response: We thank the reviewer for this important point regarding the interpretation and quantification of stress stabilization. We agree that, without clarification, the term "stabilization" could be perceived as overly qualitative.

(i) Control condition (Section 3.2 / Fig. 3). In the single-step experiments, Fig. 3 is intentionally restricted to cells for which a bleb onset time can be identified within the imaging window, because alignment of stress time series requires this reference time point. In these onset-aligned cells, we consistently observe a stereotyped sequence: traction stress increases in the frames preceding bleb onset and continues to rise around the onset itself (i.e. before and immediately after initiation), followed by a plateau and/or decline during the subsequent phase of bleb expansion over the remainder of the recording. Within the analyzed time window we do not observe a systematic secondary increase after the onset-associated rise.

Importantly, the broader statement that stresses stabilize or decline once bleb expansion happens, is supported not only by the onset-aligned subset in Fig. 3, but also by inspection of the full dataset, including cells that were already blebbing at the start of imaging due to pre-confinement. These latter cells are not included in Fig. 3 because their onset cannot be aligned, but they show the same qualitative behavior that during ongoing bleb expansion stress does not continue to increase. We have revised the *Results* to make clear that Fig. 3 illustrates the onset-centered time series, while the conclusion about stabilization/decline during bleb expansion is consistent across the full population.

To address the reviewer's request for a quantitative meaning, we now define "stabilization" explicitly as the absence of a sustained increase during the bleb-expansion phase, i.e. a near-

zero or negative trend of the stress trace over the late part of the recording (lines 273-276; section 5.10 and Fig. 2f caption).

(ii) Blebbistatin condition. We also agree that our original wording for the blebbistatin condition could be interpreted as unquantified. In blebbistatin-treated cells, imaging of the actin/cell channel was not performed because blebbistatin is strongly light-sensitive and exhibits pronounced phototoxicity under repeated fluorescence illumination. This prevents reliable identification of bleb onset times and therefore precludes an onset-aligned analysis similar to Fig. 3. We now state this limitation explicitly in the manuscript (lines 330-333).

Nevertheless, the population-averaged stress time series in Fig. 5c show that, following the single-step confinement, both lateral and normal stresses remain low and nearly constant throughout the recording, which is qualitatively distinct from the control condition in Fig. 3c. We therefore specify in the text that our conclusion for blebbistatin is based on the absence of a pronounced transient stress increase in the stress time series, rather than on an onset-aligned comparison to bleb initiation (lines 340-343).

Reviewer's comment: *"In most cells we also observed that the cortical actin network becomes visibly denser prior to bleb onset, with actin accumulation evident in the preceding frames"- this statement needs to include quantified evidence in the figures.*

Response: We thank the reviewer for raising this point. We agree that any statement about cortical actin densification prior to bleb onset should be supported by quantitative analysis. In the present experimental configuration, however, a robust quantification of cortical actin density is not straightforward. Under confinement, changes in apparent actin intensity are strongly affected by cell geometry, local thickness, axial position relative to the focal plane, and photobleaching, making fluorescence intensity an unreliable proxy for true cortical actin density without additional normalization and controls.

Because such an analysis would require a dedicated methodological framework (e.g. cortical segmentation, thickness correction, and depth-dependent intensity calibration) that goes beyond the scope of this study, we have removed the corresponding sentence from the *Results* section to avoid overinterpretation. The revised manuscript therefore focuses on force-based and onset-aligned analyses that are quantitatively supported by the presented data.

Reviewer's comment: *Presenting the individual data as heatmaps is much harder to interpret than just showing 12 individual curves to illustrate the heterogeneity observed.*

Response: We thank the reviewer for this suggestion and agree that individual trajectories can be very useful for illustrating single-cell behavior. In this case, however, we deliberately chose a heatmap representation because it allows several key aspects of the data to be visualized simultaneously in a compact and interpretable way.

Specifically, the heatmaps display (i) the timing of bleb onset for each individual cell within the imaging window, (ii) the evolution of traction stress before, during, and after bleb ini-

tiation, and (iii) the substantial cell-to-cell heterogeneity in both magnitude and temporal response. Plotting all single-cell stress curves in one graph would result in strong overlap and obscure these features, while showing each cell in a separate panel would require an additional multi-panel figure that is not feasible within the space constraints of the main manuscript.

We have therefore retained the heatmap representation and revised the figure caption to more explicitly guide the reader on how to interpret it, including how bleb onset and different response phases are encoded. We believe this representation provides the clearest overview of the population-level dynamics while still preserving single-cell information.

Reviewer's comment: *20 μM Y27632 is on the high side and introduces the potential for off-target effects like inhibition of PKN isoforms.*

Response: We thank the reviewer for this important point. We agree that 20 μM Y-27632 is on the upper end of commonly used concentrations and that off-target effects (including inhibition of PKN isoforms) should be considered. We chose 20 μM in our confinement assays because the experiment is performed in the presence of a thick PAA hydrogel, and a fraction of the added compound partitions/diffuses into the gel volume. We therefore aimed to ensure robust effective exposure of the confined cells throughout the measurement period.

To address the reviewer's concern directly, we repeated the same experiment using a lower dose of Y-27632 (10 μM). Under this condition we obtained the same qualitative and quantitative outcome as with 20 μM: blebbing was abolished and both lateral and normal stresses were strongly reduced compared to the vehicle control. This indicates that our conclusions do not depend on using the high-end concentration. We have added this information to the revised *Methods* (lines 840-841), Supplementary Figure S7, as well as in the rebuttal letter for transparency (Figure 2 of the rebuttal letter).

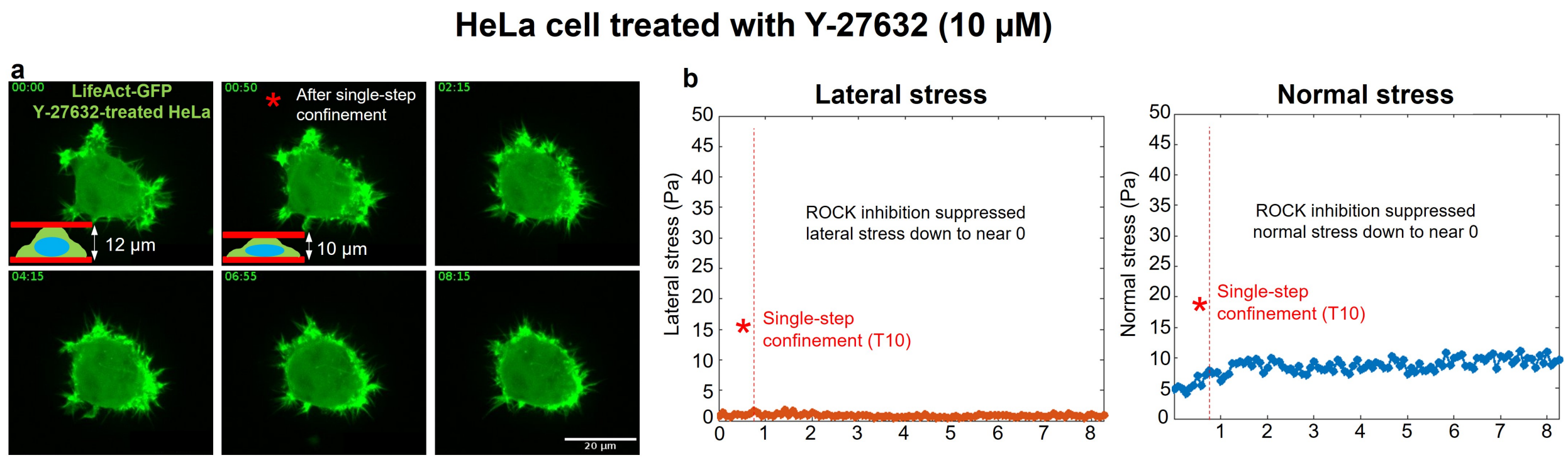

Figure 2: **Y-27632 at 10 μM reproduces the suppression of blebbing and traction stresses under single-step confinement. (a)** Representative time-lapse of a HeLa LifeAct-GFP cell during a single-step confinement from 12 μm to 10 μm, showing no bleb formation throughout the recording (scale bar, 20 μm). Consistent with this example, no blebbing was observed across other cells, including experiments at much stronger confinement (down to 3–4 μm). **(b)** Lateral (blue) and normal (orange) traction-stress time series for the same cell, remaining near zero and within the noise level over the full imaging window. In untreated controls, comparable confinement steps elicit substantially larger stress magnitudes and a detectable post-step stress increase, confirming that 10 μM Y-27632 is sufficient to strongly suppress force generation and the associated mechanical response.

Reviewer's comment: *Why does the cell in Fig. 3 get compressed from 9.5 to 8 μm, while the cell in Fig. 4 gets compressed from 11 to 6 μm? Even accounting for the variety in starting height, this wouldn't be the same level of strain. The same goes for Fig. 4, where the cell is compressed from 9 μm to 3 μm.*

Response: We thank the reviewer for this careful observation and agree that the representative examples shown in Figs. 3–5 are not strain-matched (e.g. 9.5 μm to 8 μm in Fig. 3 versus 11 μm to 6 μm in Fig. 4 or 9 μm to 3 μm in Fig. 5). This was a deliberate choice and we apologize that the rationale was not stated clearly in the previous version.

Our intention in selecting these example cells was to illustrate the qualitative contrast between conditions across a range of confinement severities, rather than to provide a one-to-one comparison at identical strain. Specifically, we chose (i) a control example showing that blebbing and elevated stresses can already occur even at relatively modest confinement, and (ii) inhibitor examples demonstrating that under much stronger confinement the measured stresses remain markedly reduced (and, for Y-27632, blebbing is suppressed), while

blebbistatin-treated cells can still bleb at high strain but without the characteristic stress time series observed in controls. These examples were therefore selected to visually emphasize that the inhibitor effects are robust and not restricted to a narrowly tuned confinement level.

Importantly, our conclusions are not based on these single examples but on population-level quantification across many cells measured over a range of initial heights and applied strains.

Reviewer's comment: *Given the central importance of blebbing to many of the conclusions made, it's unclear why no rigorous quantification of blebbing, both as a function of strain and inhibitor treatment, is provided. The only information that is given is broad statements like "blebbing was largely suppressed at low to moderate strain levels ($< 50\%$), at very high strains ($> 60\%$) a subset of cells still developed blebs". These statements are begging for accompanying graphs and statistics.*

Response: We thank the reviewer for raising this important point and agree that, given the central role of blebbing in our interpretation, a quantitative description is desirable. We would like to clarify both what is already quantified in the manuscript and why a full statistical blebbing–versus–strain analysis is not the central scope of the manuscript.

Blebbing in our experiments is not treated as a binary population-level endpoint (e.g. blebbing vs. non-blebbing), but as a *time-resolved single-cell event* that can occur before imaging, during the imaging window, or after the recording ends. As shown in the single-step experiments, more than 90% of cells exhibit blebbing either prior to the start of imaging or initiate blebbing during the recording period. For this reason, Fig. 3 and the associated analyses focus specifically on cells in which bleb onset occurs *within* the imaging window, enabling alignment of force trajectories to the time of bleb initiation. This selection is essential for the temporal force–bleb correlation central to the study. As the aim of the manuscript is to highlight the potential of the CFM with cell blebbing as an example, the details of cell blebbing as biological event are not in the focus.

Hence, we deliberately frame statements regarding blebbing frequency and strain dependence conservatively and qualitatively, while grounding our main conclusions in quantitative, time-resolved force measurements aligned to bleb onset when this information is experimentally accessible.

Reviewer's comment: *In Fig. 4C, it appears that two cells deemed outliers for average lateral stress were not deemed outliers for average normal stress (as there are four outliers on lateral and only two on normal). Given that these two quantities should be linked, it appears inappropriate to not rule out both when one is an outlier.*

Response: We thank the reviewer for this careful observation and agree that the outlier handling in Fig. 4C was potentially confusing. In the Y-27632 condition, where overall stresses are strongly reduced and we do not observe a pronounced stress build-up phase, it is

reasonable to expect that cells flagged as extreme values in one component should be treated consistently across both readouts.

We have therefore revised the analysis/figure to apply a *joint* outlier criterion at the single-cell level: if a cell is identified as an outlier in either lateral or normal average stress, it is marked as an outlier for both stresses. This removes the apparent inconsistency noted by the reviewer and does not change the conclusions of the comparison between conditions. Implementing this unified criterion also affected the number of flagged points across figures: under the updated method, the Y-27632 condition (Fig. 4C) contains only two outlier cells, and the blebbistatin condition (Fig. 5C) no longer contains any outliers. We have updated Figs. 4C and 5C accordingly and clarified the joint outlier procedure in detail in the *Methods* (lines 940-955).

Reviewer's comment: *The formatting of the results section 4.6, with bolded highlights and arrows, is not generally appropriate for publication. The Pearson correlation heatmap is unnecessary and should be a supplemental figure.*

Response: We thank the reviewer for this suggestion and agree that the formatting in Section 4.6 (now Section 3.6 in the revised manuscript) (bold highlights and arrow-based emphasis) is not ideal for a final publication format. We have revised the *Results* (section 3.6) to remove the bolded highlights/arrows and replaced them with standard scientific phrasing, while preserving the underlying quantitative conclusions.
We also agree with the reviewer that the Pearson-correlation heatmap is not essential for the main narrative of the manuscript. We have therefore moved the correlation heatmap to the Supplementary Information (Supplementary Figure 6) and adjusted the main text and figure references accordingly.

Reviewer's comment: *In the schematic in Figure 8, the nucleus has the same shape in almost every image. Is this consistent with the levels of compression applied?*

Response: We thank the reviewer for this excellent observation and insightful question. In the experiments underlying Fig. 8 we did not label the nucleus, because adding an additional fluorescence channel would have increased acquisition time and/or illumination load. This would have compromised our ability to image at the required temporal resolution (5 s per frame), which is already comparable to or slower than the fast relaxation times of some cells. Consequently, we cannot make a data-supported statement about nucleus shape dynamics in these specific experiments.

Conceptually, we agree that nuclear deformation is expected to depend on the applied strain: when cells are confined below their native height, the nucleus will generally experience increasing deformation with increasing compression. However, Fig. 8 is intended as a working model summarizing the force–bleb coupling and stress redistribution observed in our assays, rather than as a quantitative depiction of nuclear mechanics. We therefore kept the nucleus shape schematic and largely invariant across most panels to avoid implying nucleus-specific

conclusions that we did not measure. Only in the final panel illustrating very strong compression (and blebbistatin treatment) did we sketch a more deformed nucleus to convey the qualitative expectation under extreme confinement.

We have clarified this point in the figure caption to ensure that the schematic is not interpreted as a direct readout of measured nuclear deformation.

Reviewer's comment: *The authors claim in the discussion to have achieved mechanistic clarity. Two inhibitors were used, both of which result in reduced actomyosin contractility, were used at the high end of their working concentrations. This is not the generally accepted standard for mechanistic clarity, which often requires confirmatory knockouts/knockdowns, rescue experiments, etc.*

Response: We thank the reviewer for this important clarification and agree that the phrase "mechanistic clarity" can be interpreted too strongly if taken to imply definitive molecular pathway validation. We did not intend to claim full causal mechanistic proof in the sense of genetic knockouts/knockdowns, rescue experiments, or comprehensive pathway dissection.

Rather, our aim was to demonstrate *mechanistic insight at the level of cellular force generation and mechanical response* under confinement.

We fully agree that pharmacological inhibition—even using two inhibitors targeting actomyosin contractility at commonly used concentrations—does not meet the standard of molecular mechanistic proof. Accordingly, we revised the *Discussion* to soften the wording and removed phrasing that implied "mechanistic clarity". We further note that genetic and rescue-based approaches will be required in future studies to establish causal molecular mechanisms.

As this manuscript is primarily a methods-focused study, we believe that the key advance is the ability of CFM to resolve time-resolved force responses under controlled confinement, which enables such mechanistic questions to be addressed rigorously in future work.

Reviewer's comment: *It appears that a number of experiments that have been performed for previous versions of this manuscript have been left off of this one or moved to supplemental figures. A number of experimental methods, including the use of C2C12s and CT26 cells, neutrophils, cancer spheroids, drosophila, and 9/21/114 kPa hydrogels, are included in the methods but not mentioned in the main text.*

Response: We thank the reviewer for pointing this out and appreciate the opportunity to clarify. The experiments and biological systems mentioned (C2C12 and CT26 cells, neutrophils, cancer spheroids, *Drosophila* embryos, and hydrogels of different stiffnesses) were included in earlier versions of the manuscript in the main text. In the previous round of review, however, we were explicitly asked to focus the main narrative more tightly on a single, coherent application of the method and to move additional applications to the SupplementaryInformation.

We now explicitly reference these supplementary applications in the *Discussion* (lines 732-752) to make their role and scope clear, while maintaining a focused and coherent main narrative.

Reviewer #2 (Remarks on figshare data availability):

This is not data, it's supplementary figures.

Response: We thank the reviewer for pointing this out, and we apologize for the previous figshare upload. The files we provided previously were indeed supplementary figures rather than the underlying source data. We have therefore updated the figshare deposit to include the underlying numerical source data used to generate the figures. The full raw image stacks are very large and are not practical to upload in full; instead, we provide the processed numerical outputs necessary to reproduce the analyses and plots from the deposited data.

Response to Reviewers

Manuscript: *CFM: Confinement Force Microscopy—a dynamic, precise and stable microconfiner for traction force microscopy in spatial confinement*

Authors: Abbasi *et al.*

Manuscript ID: **NMETH-A53559C**

Reviewer #1

Remarks to the Author:

In this second revision of their article, the authors have addressed the minor comments I had after their first revision, which was mostly related to the discussion part of the article. I also read in detail their answer to the other reviewer, who had a very long list of comments. I think they have all been addressed satisfactorily. Most of them were related to citing relevant literature, evaluation of the device and other devices for confinement, so no serious fundamental concern remained and these points were fixed properly by the authors. Other comments were requests for clarification of aspects that were correct, in my opinion, but perhaps not fully clear as presented in the article. The authors explained these points in detail and very clearly, and made the appropriate modifications to avoid these confusions. I think Reviewer 2 pointed to all the possible sources of misinterpretation and confusion, and the authors made a great effort to improve their article. It should now be published as it is without any need for further revisions.

Response:

We sincerely thank Reviewer 1 for this highly positive, thoughtful and encouraging assessment of our revised manuscript. We are very grateful for the reviewer's careful reading of both the revised paper and our responses to the previous comments. It is especially encouraging to us that the reviewer considers the earlier concerns to have been addressed satisfactorily and recognizes the effort made to improve the manuscript's clarity, interpretation and overall presentation. We greatly appreciate these generous comments and the reviewer's conclusion that the manuscript is now suitable for publication without further revision.
